# Epithelial Gab1 Restricts Sepsis‐Induced Intestinal Injury by Orchestrating TNF/NF‐κB Axis

**DOI:** 10.1155/mi/5486971

**Published:** 2026-01-31

**Authors:** Wei Jin, Yanchuang Wu, Xiaoqing Cheng, Yu Pan, Lifeng He, Yun Xu, Jiaqi Xu, Xue Zhang

**Affiliations:** ^1^ Department of General Surgery, Sir Run Run Shaw Hospital, Zhejiang University School of Medicine, Hangzhou, 310016, China, zju.edu.cn; ^2^ Department of Pathology, Sir Run Run Shaw Hospital, Zhejiang University School of Medicine, Hangzhou, 310016, China, zju.edu.cn; ^3^ Department of Respiratory and Critical Care Medicine, Center for Oncology Medicine, The Fourth Affiliated Hospital of School of Medicine, International School of Medicine, International Institutes of Medicine, Zhejiang University, Yiwu, 322000, China, zju.edu.cn; ^4^ Department of Pathology and Pathophysiology, Department of Respiratory Medicine of Sir Run Run Shaw Hospital, Zhejiang University School of Medicine, Hangzhou, 310016, China, zju.edu.cn

**Keywords:** adaptor protein Gab1, apoptosis, intestinal epithelial cells, intestinal injury, sepsis

## Abstract

A series of intestine‐related alterations have been considered a key factor in triggering sepsis, with increased apoptosis of intestinal epithelial cells (IECs) notably contributing to this process. Compromised gut barrier due to IEC apoptosis promotes bacterial translocation and inflammatory responses, which in turn escalates to further IEC death and barrier defects. Nevertheless, the precise mechanisms that safeguard IECs from apoptosis and interrupt this vicious cycle are yet to be elucidated. Here, we report that Grb2‐associated binder 1 (Gab1) expression is diminished in the intestines of both septic patients and established sepsis models. Epithelial Gab1 deficiency rendered mice susceptible to lipopolysaccharide (LPS)‐induced sepsis by sensitizing IECs to apoptosis, thereby contributing to systemic inflammation and markedly exacerbating septic lethality. Mechanistically, Gab1 mitigated apoptotic signaling via IKKβ‐dependent NF‐κB activation and subsequent transcriptional regulation of apoptotic genes in response to TNF‐α. Collectively, our findings define a protective role for Gab1 in sepsis‐induced intestinal injury by sustaining apoptotic balance and intestinal homeostasis, which provides new insights into therapeutic strategies for sepsis management, particularly those aiming at restoring immune homeostasis and improving barrier function.

## 1. Introduction

Sepsis, a life‐threatening organ dysfunction due to dysregulated host response to infection, was estimated to cause 11 million deaths in 2017, accounting for nearly 20% of all global fatalities [1, 2]. Recent advances reveal that the gut plays an important role in sepsis pathogenesis, wherein its barrier function is recognized as a key determinant [3–5]. Intestinal epithelial cells (IECs) linked by tight junctions establish a physical and biochemical barrier, which provides the interface between the host and microorganisms to harmonize mucosal immunity from tolerance to antipathogen responses [6, 7]. However, epithelial barrier breach leads to increased intestinal permeability and bacterial translocation, thereby provoking mucosal immune defenses with subsequent cytokine production, which triggers more IEC death and barrier defects [8, 9]. This intensifies local intestinal inflammation, systemic inflammation, and even ischemia‐reperfusion injury, contributing to a vicious feedback loop in sepsis development that may progress to multi‐organ dysfunction syndrome (MODS) [10].

To maintain barrier function and tissue homeostasis, IECs undergo dynamic and continuous turnover, a process tightly regulated by the balance between cell proliferation and cell death [11, 12]. Clinical evidence robustly demonstrates that intestinal epithelial apoptosis is a consistent hallmark of sepsis, observed in human samples with sepsis or septic shock [13]. These observations are further confirmed by various murine sepsis models, including pneumonia‐induced, lipopolysaccharide (LPS)‐induced, and the cecal ligation and puncture (CLP) models [14–17].

Apoptosis is a finely turned form of programed cell death, triggered by extrinsic and intrinsic signals. Among these, tumor necrosis factor (TNF), in particular, is a potent extrinsic inducer that significantly contributes to apoptotic cascades in the inflammatory microenvironment [18]. Upon TNF‐α binding to TNFR1, several adapter signaling proteins are recruited, including Fas‐associated death domain protein (FADD) and TNF Receptor‐Associated Factor 2 (TRAF2) [18, 19]. FADD recruits procaspase‐8, initiating a self‐amplifying caspase cascade that culminates in the activation of executioner caspase‐3, ultimately leading to apoptosis [20]. Meanwhile, TRAF2 stimulation is thought to activate MAPK, JNK, and NF‐κB signaling, thereby driving the transcriptional regulation of inflammatory and anti‐apoptotic genes [21]. Genetic studies reveal that mice with genetic ablation of *Tnfr1*, *IKKβ*, or cathelicidin (*Cnlp*) in IECs exhibit exacerbated sepsis and increased mortality owing to sensitizing cells to apoptosis [15, 16, 22]. Conversely, blocking sepsis‐induced apoptosis significantly improves survival, with transgenic mice overexpressing anti‐apoptotic protein Bcl‐2 in IECs exhibiting a survival advantage in both *Pseudomonas aeruginosa* pneumonia‐induced and CLP‐induced sepsis [14, 23–25]. Moreover, elevated caspase‐3 activation has been clinically reported in patients with sepsis or shock [13, 26]. Nevertheless, how IEC apoptosis is negatively regulated during sepsis development remains elusive. Therefore, dissecting this negative regulation of apoptosis is crucial for maintaining intestinal barrier homeostasis and an optimal immune response.

Grb2‐associated binder 1 (Gab1) is a pivotal adaptor protein that operates downstream of various receptors for growth factors and cytokines, including insulin receptor, EGFR, c‐met, and gp130 [27–29]. By forming protein complexes, Gab1 amplifies and mediates downstream signals, consequently regulating diverse biological events including cell proliferation, differentiation, and development. Global Gab1 knockout (Gab1^-/-^) mice exhibit lethality between e12.5 and e18.5, manifesting severe developmental defects across multiple organs [30]. Recent studies in adult animals revealed that Gab1 plays a crucial role in modulating acute and chronic tissue damage, inflammation, and tissue remodeling, notably in conditions such as acute lung injury (ALI), colitis, asthma, atherosclerosis, and organ fibrosis [31–35]. Additionally, cardiac‐specific Gab1 knockout mice develop heart failure associated with elevated cardiomyocyte apoptosis and impaired MAPK signaling [36]. Despite this, the pathophysiological roles of intestinal Gab1 in mediating cell death during sepsis‐induced intestinal injury and its contribution to septic lethality remain unknown.

In this study, we revealed that *Gab1* was downregulated in the intestines of both septic patients and established sepsis models. Gab1 deficiency in IECs enhances susceptibility to sepsis‐induced intestinal injury and subsequently leads to increased septic lethality in mice, owing to uncontrolled epithelial cell apoptosis. Mechanistically, Gab1 orchestrates apoptotic balance by associating with IKKβ to positively regulate NF‐κB activation and the subsequent transcription of pro‐ or anti‐apoptotic genes. Taken together, these findings shed light on the critical role for Gab1 in IEC apoptosis and septic intestinal injury, thereby providing new insights for therapeutic strategies in sepsis.

## 2. Materials and Methods

### 2.1. Human Samples

Clinical intestinal tissues collected from patients with abdominal sepsis complicated by enterocutaneous fistula were obtained from Sir Run Run Shaw Hospital, Zhejiang University School of Medicine, China. A total of 21 participants were enrolled, including a control group, a sepsis group (non‐CD sepsis), and a CD‐induced sepsis group (*n* = 7 per group). The diagnosis of non‐CD sepsis or CD‐induced sepsis was based on routine laboratory tests, clinical characteristics, radiological criteria, endoscopic examination, and histological analysis. Septic intestinal tissues were obtained from these patients under surgical resection. Control groups were recruited based on their medical history and routine laboratory tests; their normal ileal and colonic tissues were verified via endoscopic and histological examination. Detailed demographic information for the participants is provided in Supporting Information 1: Table S1. This retrospective study was conducted in accordance with the Declaration of Helsinki, and approved by the Medical Ethics Committee of Sir Run Run Shaw Hospital (approval ID: 2025‐2521‐01).

### 2.2. IHC Staining and Score

Human intestinal samples underwent standardized preparation, including fixation in 4% formalin, paraffin‐embedding, and subsequent sectioning into 4 μm sections. Following deparaffinization and rehydration, antigen retrieval was performed on paraffin sections in 10 mM, pH 6.0 citric acid, after which they were stained with primary antibody Gab1 (Abcam, ab59362) or Gab2 (Abcam, ab235932). Semi‐quantitative scoring of integrated staining intensity and positive cell percentage was performed using Constantine’s protocol. Staining intensity was assessed as follows: 0 = no color; 1 = yellow; 2 = brown to yellow; and 3 = brown. The proportion of positive cells was graded: 0 = less than 10% positive cells; 1 = between 10% and 40% positive cells; 2 = between 40% and 70% positive cells; 3 = at least 70% positive cells. The intensity and proportion scores were then added up.

### 2.3. Mice

Gab1^f/f^ mice were generous gifts from Dr. Gen‐Sheng Feng (Department of Pathology, Division of Biological Sciences and Moores Cancer Center, UCSD). Gab1^f/f^ mice were crossed with Villin‐Cre (Vil1‐iCre) mice on a C57BL/6 background (GemPharmatech, #T00471) to obtain IEC‐conditional Gab1‐knockout mice (Villin‐Cre Gab1^flox/flox^, Gab1^IEC-KO^) and their littermate controls (Gab1^flox/flox^, Gab1^f/f^). The Gab1‐flox allele was detected by PCR using the following primers: Forward: 5’‐ GGTGAATCGACGGGTGCTTGTGA‐3’ and Reverse: 5’‐ CAGATTGGCCTTGAACTGGTAAG‐3’. The Villin‐Cre transgenic allele was detected using the following primers: Forward: 5’‐GGGCAGTCTGGTACTTCCAAGCT‐3’ and Reverse: 5’‐AGTTTCCAAACTCCAGGTGACAGG‐3’. Mice were housed in a specific pathogen‐free facility at Laboratory Animal Center of Zhejiang University. All mice were maintained on a 12‐h light/12‐h dark cycle in a temperature‐ and humidity‐controlled environment (25°C, 50% humidity) and fed with sufficient water and food. All animal experiments were performed according to protocols approved by the Institutional Animal Care and Use Committee of Zhejiang University School of Medicine (approval ID: ZJU20250082).

### 2.4. Induction of Sepsis Model

Eight‐ to 10‐week‐old Gab1^IEC-KO^ mice and their male littermates, weighing ~25 g, were challenged with a dose of LPS (5 mg/kg BW; Sigma–Aldrich) in sterile saline via intraperitoneal injection. After 24 h, mice were humanely euthanized by carbon dioxide inhalation and then sacrificed. Intestinal tissues were then collected for further study including pathological examination. For survival analyses, mice were injected intraperitoneally with LPS (10 mg/kg BW) and the survival rate of mice was monitored every 12 h.

### 2.5. Cells

The human cell line HT29 (colorectal cancer, ATCC HTB‐38, RRID CVCL_0320) was obtained from American Type Culture Collection (ATCC) and cultured in RPMI 1640 medium (Gibco) supplemented with 10% fetal bovine serum (FBS) (Gibco), penicillin (100 U/mL), and streptomycin (100 μg/mL) (Gibco) at 37°C and 5% CO_2_. The human cell line HEK293T (embryonic kidney; ATCC CRL‐3216, RRID CVCL_0063) was obtained from ATCC and maintained in DMEM/high glucose (Gibco) supplemented with 10% FBS (Gibco), penicillin (100 U/mL), and streptomycin (100 μg/mL) at 37°C and 5% CO_2_. The identity of both the HT29 and HEK293T cell lines was confirmed by short tandem repeat (STR) analysis with a> 95% match to the ATCC database. The cells were confirmed not to be misidentified or contaminated, including mycoplasma contamination. To construct Gab1‐knockdown HT29 cell line, shGab1 cDNA were constructed into the PLKO1 puro vector (Addgene plasmid #8453) and subsequently packaged into lentiviruses in 293T cells using Lipofectamine 3000 Kit (Thermo Fisher Scientific). HT29 cells were infected with shGab1 lentivirus and then screened by puromycin. For stimulation, cells were plated on 6‐well plates at 5 × 10^5^ cells per well overnight and then stimulated with TNF‐α (Novoprotein) for indicated time. Single cell suspension or cell lysates were further analyzed by flow cytometry, immunofluorescence staining and Western blot.

### 2.6. Flow Cytometry Analysis

Single cell suspension was prepared as previously described. Live/dead cell and apoptotic cell discrimination done using 7‐AAD and Annexin V staining (BioLegend) according to manufacturer’s protocols. Flow cytometry was performed using NovoCyte flow cytometer (ACEA) and analyzed with FlowJo software.

### 2.7. Quantitative PCR (qPCR)

Total RNA was extracted using TRIzol reagent (Pufei Biology) and reverse‐transcribed to cDNA using the ReverTraAce qPCR RT kit (Toyobo). qPCR was performed on the Light‐Cycler Roche 480 (Roche) using SYBR Green Kit (Vazyme Biotech). The mRNA expression was calculated using the equation RQ = 2^−ΔΔCt^ method and normalized to β‐actin. The primers used for qPCR are listed in Supporting Information 1: Table S2.

### 2.8. Immunoblotting

Cell lysates were prepared and protein concentration was determined by BCA Protein Assay kit (Thermo Fisher Scientific). Samples were separated by SDS‐PAGE gels and transferred onto nitrocellulose membranes (Pall). The membranes were then incubated overnight at 4°C with primary antibodies including Gab1 (Proteintech, 26200‐1‐AP, 1:1000), phospho‐p65 (Ser536) (CST, 3033, 1:1000), p65 (CST, 8242, 1:1000), phospho‐ERK (Thr202/Tyr204) (CST, 4370, 1:1000), ERK1/2 (CST, 4695, 1:1000), phospho‐p38 (Thr180/Tyr182) (CST, 4511, 1:1000), p38 (Proteintech, 14064‐1‐AP, 1:1000) cl‐Caspase3 (CST, 9664, 1:1000), Caspase3 (CST, 9662, 1:1000), cl‐Caspase8 (CST, 9496, 1:1000), Caspase8 (CST, 4790, 1:1000), Bcl‐2 (Proteintech, 12789‐1‐AP, 1:1000), Bax (Proteintech, 50599‐2‐Ig, 1:1000), Bcl‐XL (Proteintech, 10783 1‐AP, 1:1000), phospho‐IKKα/β (Ser176/180) (CST, 2697, 1:1000), IKKα(CST, 61294, 1:1000), IKKβ(CST, 8943, 1:1000), β‐actin (Huabio, M1210‐2, 1:2000), GAPDH (Huabio, ET1601‐4, 1:2000), β‐tubulin (Proteintech, 10094‐1‐AP, 1:2000), Lamin B1 (Proteintech, 12987‐1‐AP, 1:1000), followed by IRDye 680/800 secondary antibodies (LI‐COR).

### 2.9. *H*istopathology

Colons and ilea from euthanized mice with or without LPS treatment were excised and then flushed by PBS. The intestine tissues were fixed, embedded in paraffin, and sectioned to 4 μm slices for H&E, PAS and Alcian blue staining. MPO staining was performed on formalin fixed tissues using established protocol. Paneth cell was stained with anti‐lysozyme antibody (abcam, ab108508) as previously described.

### 2.10. TUNEL Assay

Paraffin‐embedded sections derived from intestine tissues were subjected to a deparaffinization process utilizing xylene, followed by a series of rehydration procedure. In order to assess IEC death, TUNEL assay was performed with In Situ Cell Death Kit (Roche) according to the manufacturer’s instructions. Sections were then stained with DAPI and analyzed by a confocal microscope (FV3000, Olympus). The images were further processed using Olympus FluoView FV31S‐SW.

### 2.11. NF‐κB Luciferase Reporter Assay

HEK293T cells were cotransfected with pNF‐κB‐Luc, pRL‐TK, and Scr/shGab1 shRNA. Luciferase activities were measured using the Dual‐Luciferase Reporter Assay System (Promega) on a GloMax luminometer (Promega) according to the manufacturer’s instructions. Firefly luciferase activity was normalized to the Renilla internal control.

### 2.12. Quantification and Statistical Analysis

Statistical analysis was performed using GraphPad Prism software (version 8.0) and presented as mean ± SEM. Two‐tailed unpaired Student’s *t*‐tests were used for comparisons between two groups. Multiple group comparisons were conducted by one‐way ANOVA followed by Tukey’s multiple comparisons tests. The log‐rank tests were performed to compare the survival rates between groups. *p* < 0.05 was considered statistically significant.

## 3. Results

### 3.1. Gab1 Expression is Decreased in the Intestine During Sepsis

Gram‐negative bacteria containing LPS in the outer membrane are the leading cause of sepsis overall [37], while Group B *Streptococcus* (GBS) exposure is the predominant cause of neonatal and infant sepsis worldwide [38]. To establish the correlation of Gab1 with sepsis progression, we analyzed a series of datasets from intestinal tissues of mice challenged with LPS or GBS. The results revealed that *Gab1* expression was significantly reduced in the intestinal tissues of septic mice compared with the controls (Figure 1A,B). Severe sepsis is also a common and clinical syndrome of acute infection and inflammation, which has been reported in both ulcerative colitis (UC) and Crohn’s disease (CD) patients [39]. Therefore, *Gab1* expression was examined in intestinal mucosa from patients with UC and CD by utilizing the human sample datasets. We observed that *Gab1* was also decreased in UC and CD patients compared with healthy controls (Figure 1C,D).

Figure 1Gab1 is down‐regulated in sepsis‐induced intestinal injury. (A) *Gab1* mRNA expression in intestinal tissues from LPS‐challenged mice (*n* = 8) and control mice (*n* = 8), revealed by analyzing a database of RNA‐Seq data (GEO accession number GSE266246). (B) *Gab1* mRNA expression in mouse intestines with (*n* = 5) or without (*n* = 4) a 4‐day GBS exposure were measured. Data were collected from GEO database GSE230856. (C) Expression of *Gab1* was analyzed using RNA‐Seq data of colonic biopsy samples isolated from patients with UC (*n* = 87) and normal controls (*n* = 21) (GEO accession number GSE87466). (D) Expression of *Gab1* was evaluated using RNA‐Seq data of intestinal biopsy samples obtained from patients with CD (*n* = 59) and normal controls (*n* = 22) (GEO accession number GSE75214). (E) *Gab1* expression in IECs isolated from mouse intestine was measured in LPS‐challenged (*n* = 3) or control group (*n* = 3). Data were collected from GEO database GSE139903. (F) *Gab1* expression in intestinal tissues was measured following administration of 0.1 mg/kg LPS, 10 mg/kg LPS, or solvent control. *n* = 3 for each group. Data were collected from GEO database GSE180795. (G) Representative IHC staining of Gab1 in intestines from patients with sepsis (non‐CD sepsis) or CD‐induced sepsis, and normal controls. *n* = 7 for each group. Scale bars 2.5 mm (overview) and 200 μm (magnification). Quantitative data are shown as mean ± SEM. Statistical significance was assessed by using 2‐tailed Student’s *t* test (A–E) and 1‐way ANOVA with multiple comparisons test (F–G);  ^∗^ *p* <  0.05,  ^∗∗^ *p* <  0.01,  ^∗∗∗^ *p* <  0.001,  ^∗∗∗∗^ *p* <  0.0001. CD, Crohn’s disease; GBS, Group B *Streptococcus*; GEO, Gene Expression Omnibus; IECs, intestinal epithelial cells; LPS, lipopolysaccharide; UC, ulcerative colitis.

**Figure:**
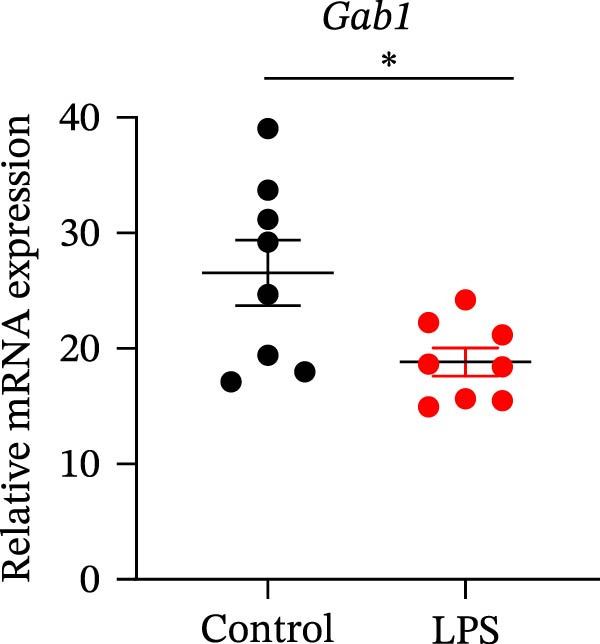
(A)

**Figure:**
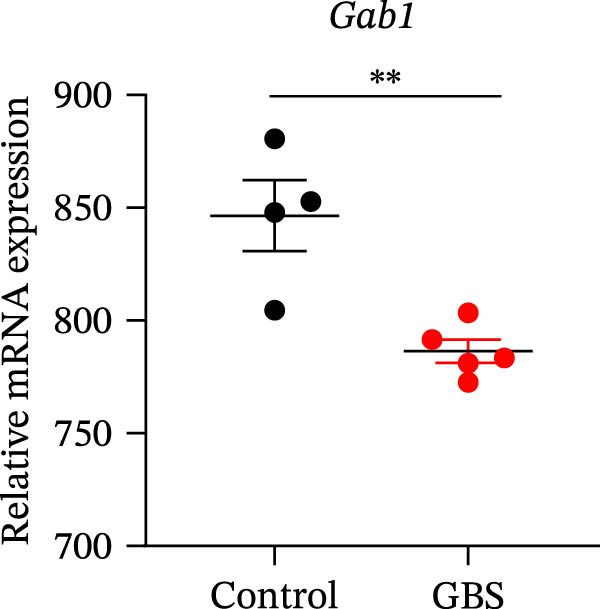
(B)

**Figure:**
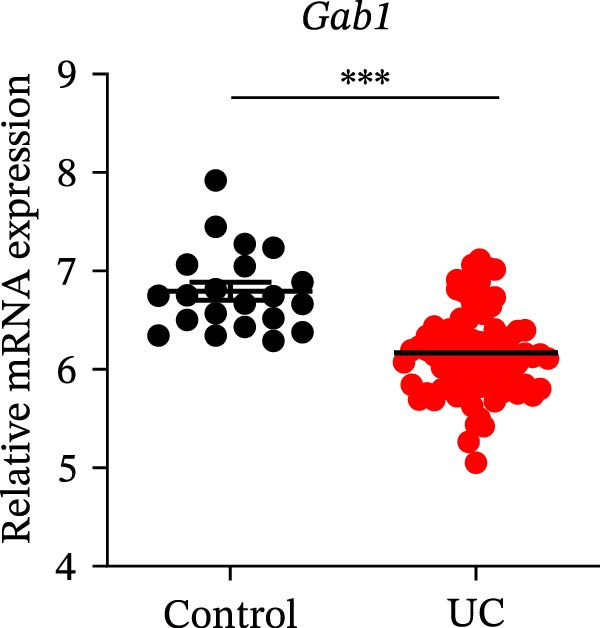
(C)

**Figure:**
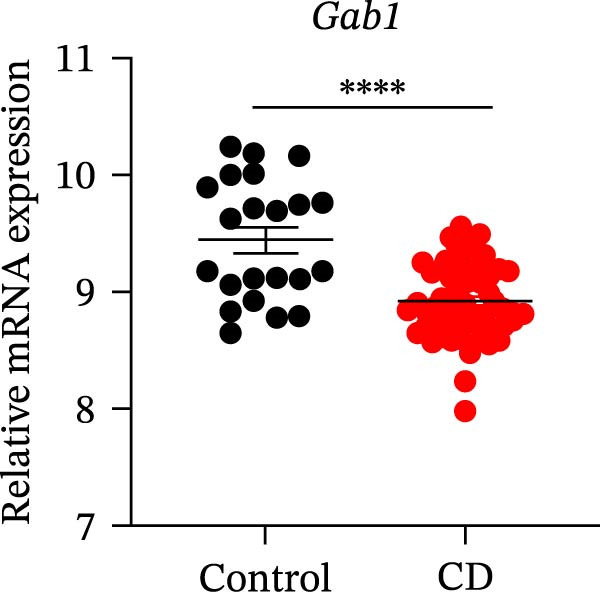
(D)

**Figure:**
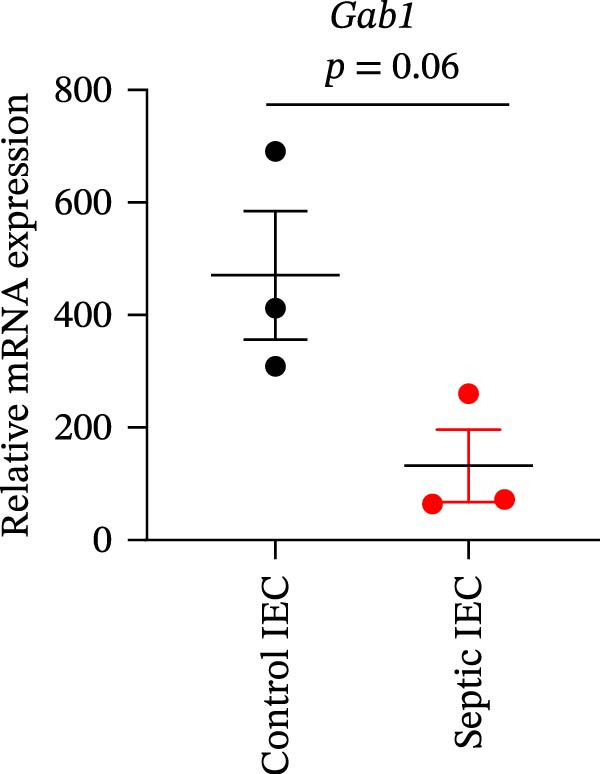
(E)

**Figure:**
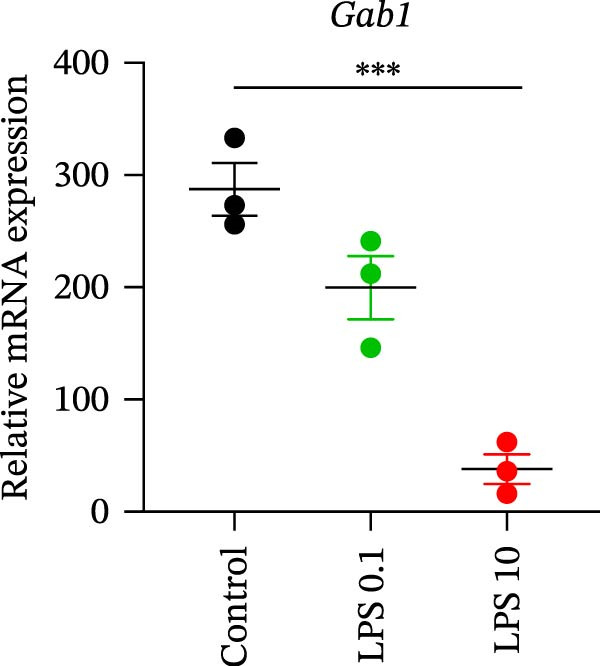
(F)

**Figure:**
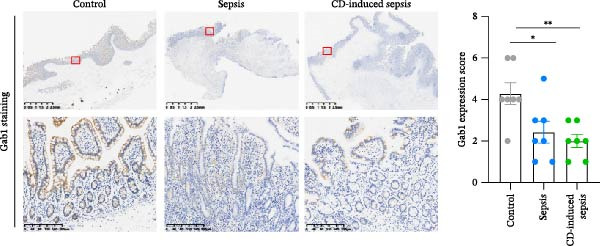
(G)

We extended our investigation to identify the cell type responsible for the reduction of Gab1 by analyzing an RNA‐sequencing dataset. The results showed a decreasing trend of *Gab1* in IECs during sepsis (Figure 1E). *Gab1* mRNA level was markedly lower in mice administered with high‐dose LPS compared to those receiving low‐dose LPS (Figure 1F), which indicated that *Gab1* expression was negatively correlated with the severity of sepsis. Next, we conducted further experiments on clinical samples of intestinal tissue from abdominal sepsis patients and control group. Both abdominal sepsis patients, comprising both non‐CD sepsis and CD‐induced sepsis cases, were complicated by enterocutaneous fistula. Immunostaining analysis revealed that Gab1 was predominantly expressed in the intestinal epithelium, and notably, its expression was significantly reduced in patients with abdominal sepsis compared to the control group (Figure 1G), whereas the levels of its homologous family protein, Gab2, remained comparable among the groups (Supporting Information 2: Figure S1). Collectively, these findings suggest a potential role for epithelial Gab1 in mucosal inflammation and intestinal damage during sepsis.

### 3.2. Gab1 Deficiency Does not Alter Intestinal Structure, IEC Subtypes, or Microbiota in Mice

To investigate the in vivo role of epithelial Gab1 in sepsis, we generated mice with conditional knockout of Gab1 in intestinal epithelium (Gab1^IEC-KO^). Gab1^IEC-KO^ mice showed no gross structural or cellular irregularities as determined by H&E staining of ileum and colon compared with their littermate controls (Figure 2A,E). Quantitative analysis further confirmed that villus length in the ileum and crypt length in the colon were comparable between the two groups (Figure 2D,G). Also, staining for several markers revealed no significant difference in epithelial cell subtypes, including Paneth cells (Figure 2B) and goblet cells (Figure 2C,F). These observations were further supported by quantification of lysozyme IHC scores and cell counts, respectively (Figure 2D,G). Additionally, the proliferation of IECs, as assessed by Ki67‐positive cells in the crypts, was similar between Gab1^IEC-KO^ and control mice (Figure 2H,I).

Figure 2No detectable changes in intestinal architecture, IEC populations, or microbiota in Gab1‐deficient mice. (A) Representative images of H&E‐stained ileum from Gab1^IEC-KO^ mice and Gab1^f/f^ littermates. *n* = 3 for each group. Scale bars, 100 μm (overview) and 50 μm (magnification). (B) Representative IHC staining for Lysozyme in ileal sections from Gab1^IEC-KO^ mice and littermate controls. *n* = 3 for each group. Scale bars, 100 μm (overview) and 50 μm (magnification). (C) Representative images of Alcian blue staining of ileal sections from Gab1^IEC-KO^ mice and littermate controls. *n* = 3 for each group. Scale bars, 100 μm (overview) and 50 μm (magnification). (D) Quantification of the villus height (A), lysozyme IHC score (B), and number of goblet cells (C) in the ileum was performed by averaging 3–5 fields per mouse. (E) Representative images of H&E‐stained colon from Gab1^IEC-KO^ mice and littermate controls. *n* = 3 for each group. Scale bars, 100 μm (overview) and 50 μm (magnification). (F) Representative PAS staining of colonic sections from Gab1^IEC-KO^ mice and littermate controls. *n* = 3 for each group. Scale bars, 100 μm (overview) and 50 μm (magnification). (G) Quantification of the crypt height (E) and number of goblet cells (F) in the colon. Data were obtained by averaging 3–5 fields per mouse. (H, I) Representative immunofluorescence images (H) and quantification (I) of Ki67‐positive cells (green) in colonic sections. *n* = 3 for each group. Scale bars, 100 μm. Quantification of Ki67‐positive cells per crypt was performed by averaging 3–5 fields per mouse. (J) Venn diagram of Gab1^f/f^ (WT) and Gab1^IEC-KO^ (KO) groups showing unique and shared OTUs for bacterial sequences based on normalized sequences. *n* = 3 for each group. (K) Analysis of α‐diversity (observed_features) in Gab1^f/f^ and Gab1^IEC-KO^ groups. *n* = 3 for each group. (L) Principal Coordinates Analysis (PCoA) showing beta‐diversity based on weighted UniFrac distances between Gab1^f/f^ and Gab1^IEC-KO^ groups. *n* = 3 for each group. (M) Relative abundances of the top 10 bacteria at the phylum level in Gab1^f/f^ and Gab1^IEC-KO^ mice were assessed using 16S rRNA amplicon sequencing. *n* = 3 for each group. Quantitative data are shown as mean ± SEM. Statistical significance was assessed by using 2‐tailed Student’s *t* test, *p* < 0.05 was considered statistically significant. ns, not significant; OTUs, operational taxonomic units; PAS, periodic acid‐Schiff; PCoA, principal coordinates analysis.

**Figure:**
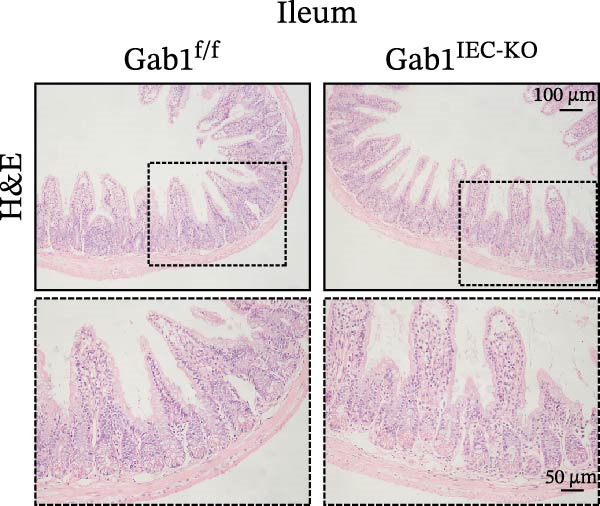
(A)

**Figure:**
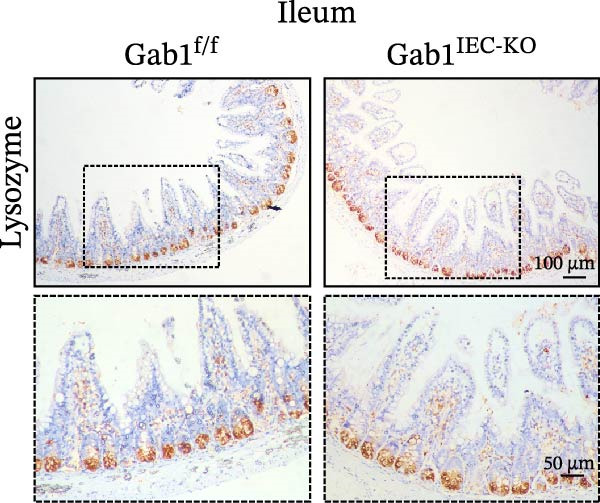
(B)

**Figure:**
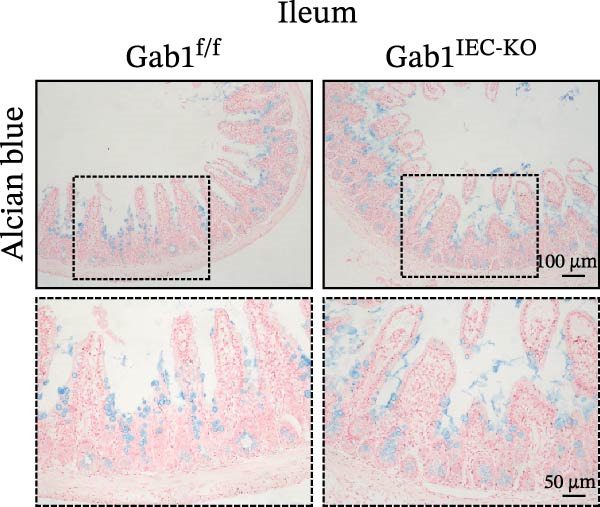
(C)

**Figure:**
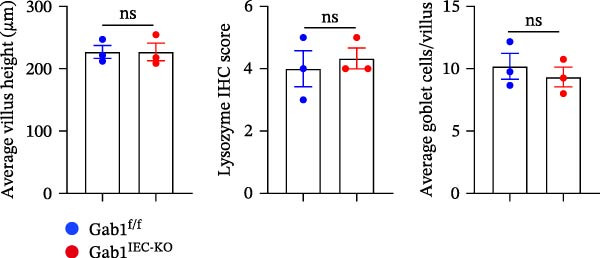
(D)

**Figure:**
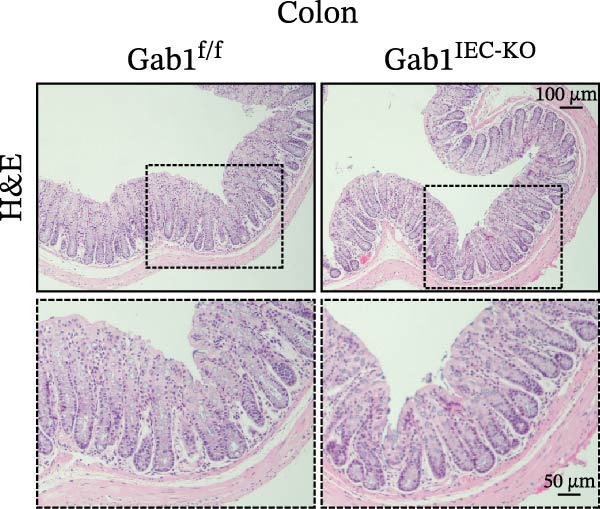
(E)

**Figure:**
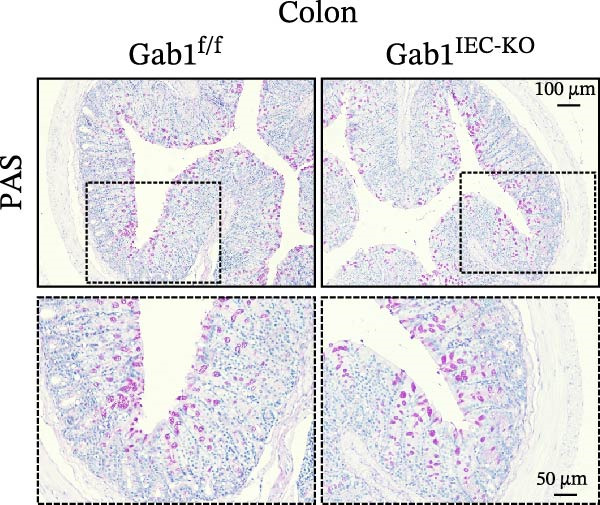
(F)

**Figure:**
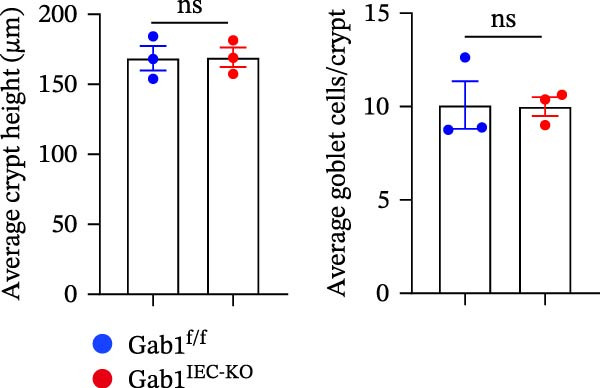
(G)

**Figure:**
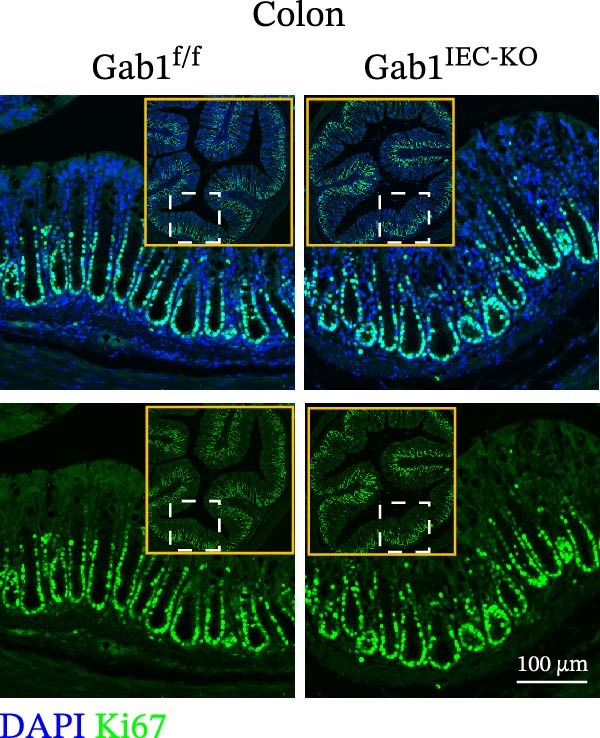
(H)

**Figure:**
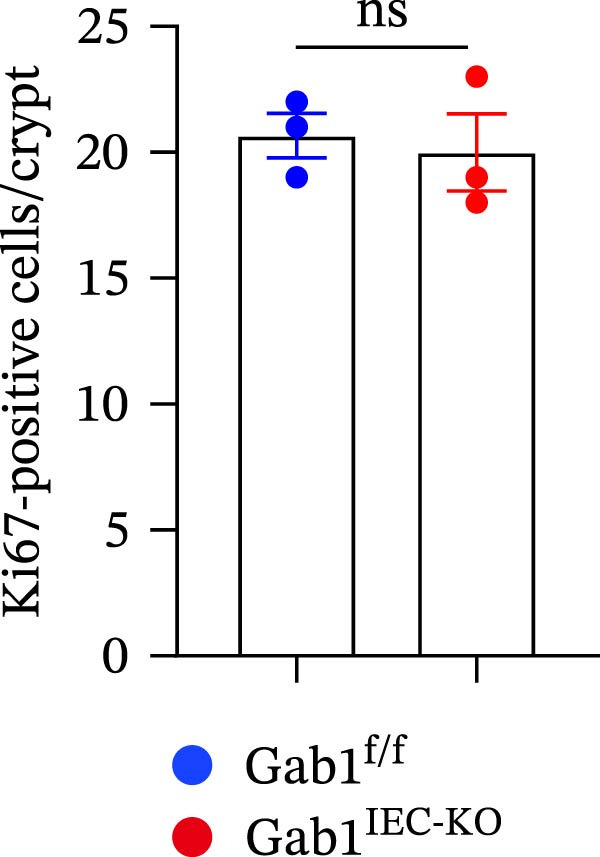
(I)

**Figure:**
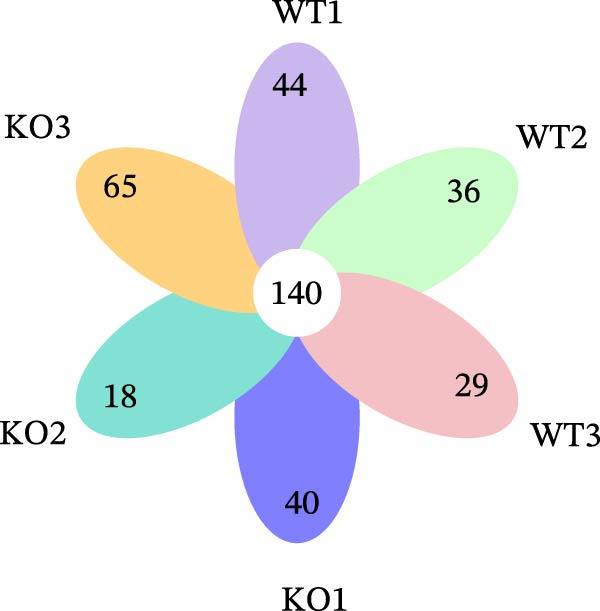
(J)

**Figure:**
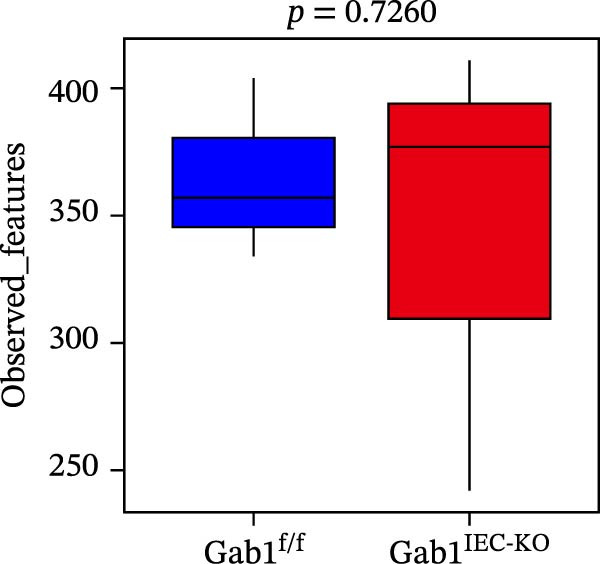
(K)

**Figure:**
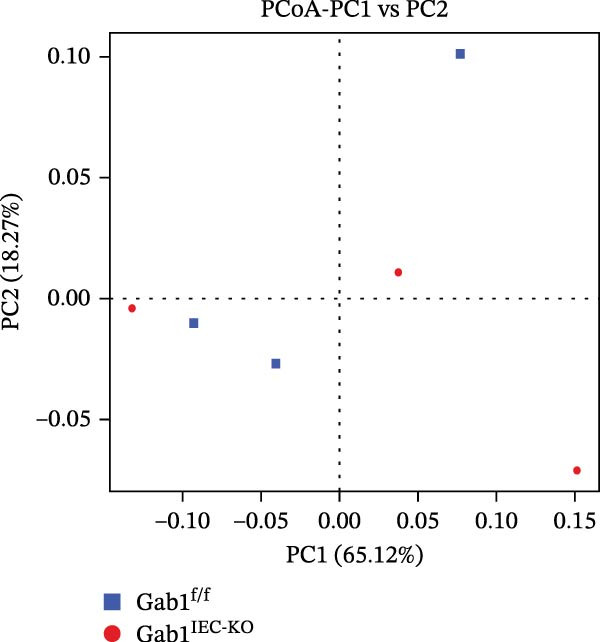
(L)

**Figure:**
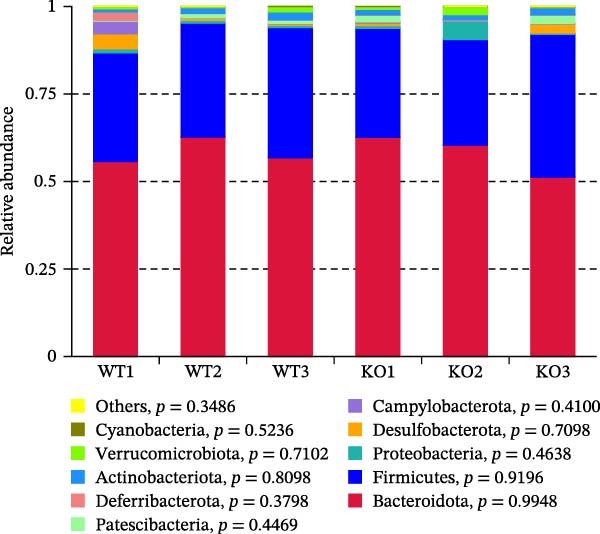
(M)

Preclinical study demonstrates a balanced gut microbiota enhances host immunity against pathogens, while its disruption increases sepsis susceptibility [5, 40]. To determine if Gab1 affects gut microbiota and thus contributes to sepsis, we analyzed the fecal microbiota composition in Gab1^IEC-KO^ mice and Gab1^f/f^ littermates by 16S rRNA sequencing. As shown in Figure 2J,M, OTU clustering and phylum‐level bacterial relative abundance analyses revealed no significant difference in species composition between Gab1^IEC-KO^ mice and Gab1^f/f^ littermates. Similarly, analysis of microbial community diversity (Figure 2K) and overall community composition (Figure 2L) showed no significant variations between the Gab1^IEC-KO^ and control groups.

### 3.3. Loss of Gab1 in IECs Predisposes Mice to LPS‐Induced Sepsis

Then, we established a toxemia model through lethal intraperitoneal LPS injection in mice, with survival monitored at 12‐h intervals. Compared with control littermates, Gab1^IEC-KO^ mice displayed significantly reduced survival rate following LPS challenge (Figure 3A). Subsequently, experimental sepsis was induced by administering LPS (5 mg/kg BW), with analyses performed 24 h post‐injection. Upon LPS treatment, Gab1^IEC-KO^ mice exhibited exacerbated histopathological features in both the ileum and colon, including extensive epithelial damage, pronounced inflammatory cell infiltration, and severe crypt architectural disruption relative to littermate controls (Figure 3B,C). Consistent with H&E staining, Gab1 deficiency in IECs amplifies mucosal inflammatory responses as evidenced by upregulated expression of key pro‐inflammatory cytokines (*Il1b*, *Il6*, and *Tnf-a*) and the chemokine *Ccl2* (Figure 3D), along with an increased infiltration of MPO‐positive cells observed in the intestines of Gab1‐knockout septic mice (Figure 3E,F).

Figure 3Epithelial Gab1 deficiency enhances susceptibility to LPS‐induced sepsis in mice. (A) Gab1^f/f^ (*n* = 11) and Gab1^IEC-KO^ (*n* = 6) mice were challenged with LPS (10 mg/kg BW), and the survival of mice was monitored. (B–F) Gab1^f/f^ and Gab1^IEC-KO^ mice were injected intraperitoneally with LPS (5 mg/kg BW) and sacrificed after 24 h. (B, C) Representative images of H&E‐stained ileum (B) or colon (C) from Gab1^IEC-KO^ mice and littermate controls following intraperitoneal administration of LPS, with histopathological score shown on the right. *n* = 6 for each group. Scale bars, 200 μm (overview) and 100 μm (magnification). (D) Quantitative real‐time PCR analysis for the mRNA expression of pro‐inflammatory cytokines (*Il1b*, *Il6*, and *Tnf-a*) and chemokines (*Ccl2*) in intestinal tissues from Gab1^f/f^ and Gab1^IEC-KO^ mice after LPS administration. *n* = 5 for each group. (E, F) Representative images of MPO staining in ileum (E) or colon (F) from Gab1^IEC-KO^ mice and littermate controls after LPS treatment. *n* = 6 for each group. Scale bars 100 μm (overview) and 50 μm (magnification). Quantification of MPO‐positive cells was performed by averaging 3–5 fields per mouse. Data are shown as mean ± SEM and are representative of three independent experiments. Statistical significance was assessed by using log‐rank test (A) and 2‐tailed Student’s *t* test (B–D);  ^∗^ *p* < 0.05,  ^∗∗^ *p* < 0.01,  ^∗∗∗^ *p* < 0.001.

**Figure:**
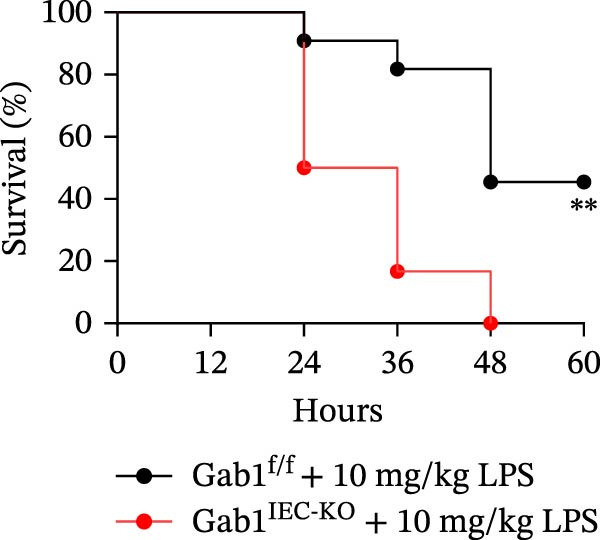
(A)

**Figure:**
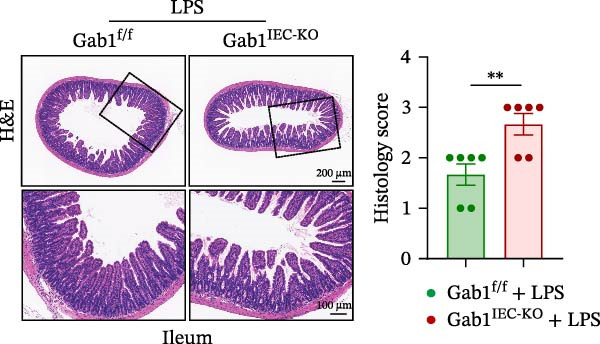
(B)

**Figure:**
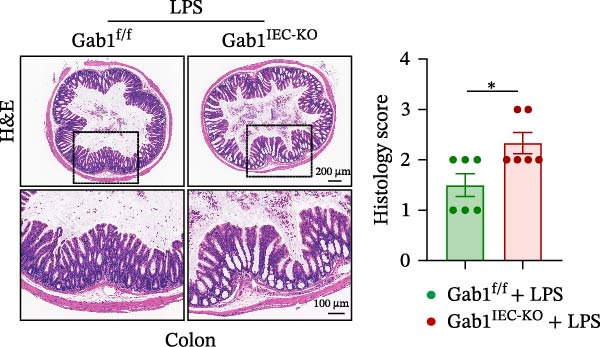
(C)

**Figure:**
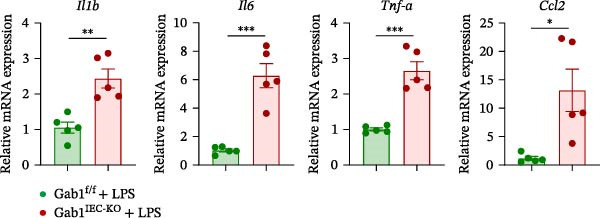
(D)

**Figure:**
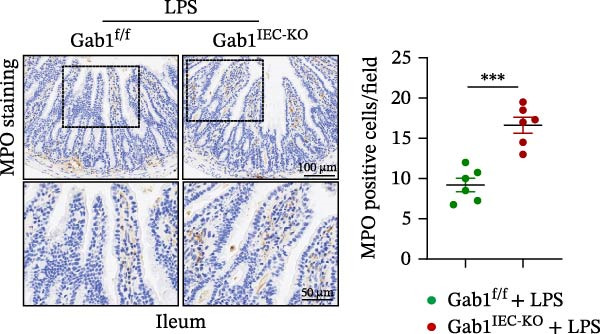
(E)

**Figure:**
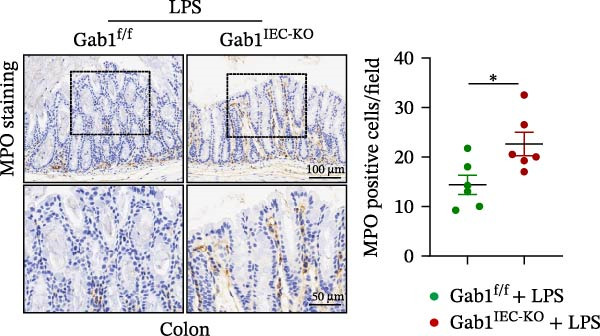
(F)

Next, the integrity of the intestinal barrier was also assessed. Immunofluorescence staining revealed that the expression and distribution of tight junction proteins ZO‐1 and Claudin‐1 were significantly reduced and disorganized in the ileum and colon of Gab1‐deficient septic mice (Figure 4A,B), indicating a profound compromise of intestinal barrier integrity conferred by Gab1 deficiency. Dysregulated apoptosis in intestinal epithelium is a key contributor to sepsis pathology. It leads to compromised epithelial barrier, promoting gut dysbiosis and enabling the systemic spread of pathogens, which is commonly observed both in patients with sepsis and in sepsis mouse models. Given this, we next evaluated IEC apoptosis in our model using the TUNEL assay. Compared with Gab1^f/f^ littermates, TUNEL‐positive epithelial cells were dramatically increased in Gab1‐KO crypts upon LPS treatment (Figure 4C,D). Consistently, Western blot analysis showed a significant decrease in Bcl‐2/Bax ratio within the intestinal mucosa of Gab1‐deficient mice (Figure 4E,F), further confirming the pro‐apoptotic effect resulting from Gab1 depletion. Collectively, these data demonstrate that IEC apoptosis triggered by Gab1 deficiency aggravates intestinal barrier dysfunction and bowel inflammation, thereby rendering mice more susceptible to LPS‐induced sepsis.

Figure 4Loss of Gab1 in IECs aggravates LPS‐induced barrier dysfunction and epithelial apoptosis in vivo. Gab1^f/f^ and Gab1^IEC-KO^ mice were challenged with LPS (5 mg/kg, i.p.), and intestinal samples were subsequently collected 24 h post‐injection. (A) Immunofluorescence staining of the tight junction protein ZO‐1 (green) and DAPI (blue) in ileal or colonic sections from the mice (*n* = 5) treated with LPS for 24 h, with quantitative data shown on the right. Scale bars 100 μm. (B) Immunofluorescence staining of the tight junction protein Claudin‐1 (red) and DAPI (blue) in ileal or colonic sections from the mice (*n* = 5) treated with LPS for 24 h, with quantitative data shown on the right. Scale bars, 50 μm. (C, D) Representative images of TUNEL staining (green) in ileal (C) or colonic sections (D) from Gab1^IEC-KO^ mice and littermate controls after LPS treatment. Scale bars, 100 μm. *n* = 5 for each group. (E, F) Representative Western blots of Bcl‐2 and Bax protein expression (E) and quantification of the Bcl‐2/Bax ratio (F) in the intestinal mucosa of Gab1^f/f^ and Gab1^IEC-KO^ mice after LPS administration (*n* = 3 per group). β‐actin was used as a loading control. Results are representative of three independent experiments. Quantitative data are shown as mean ± SEM. Statistical significance was assessed by using 2‐tailed Student’s *t* test;  ^∗^ *p* < 0.05,  ^∗∗^ *p* < 0.01.

**Figure:**
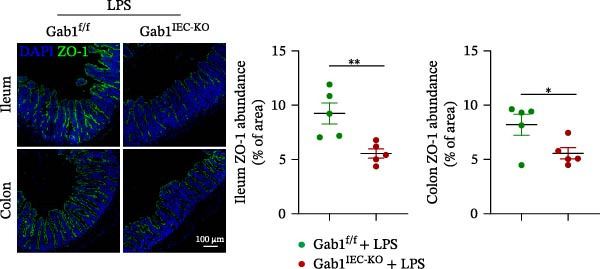
(A)

**Figure:**
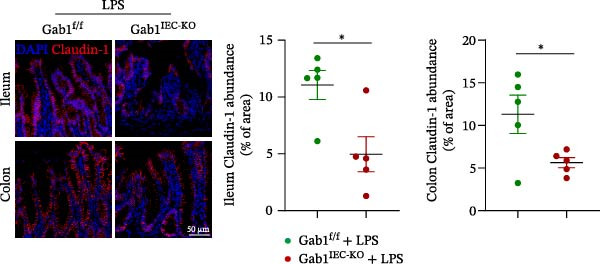
(B)

**Figure:**
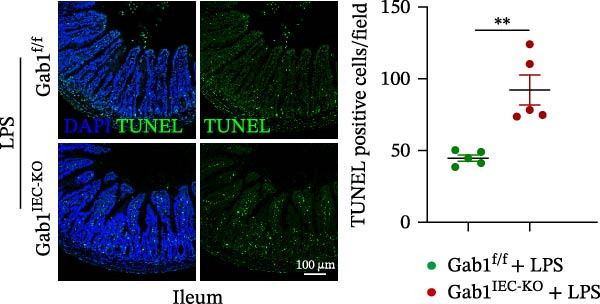
(C)

**Figure:**
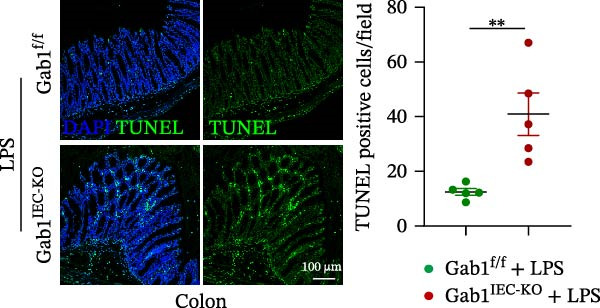
(D)

**Figure:**
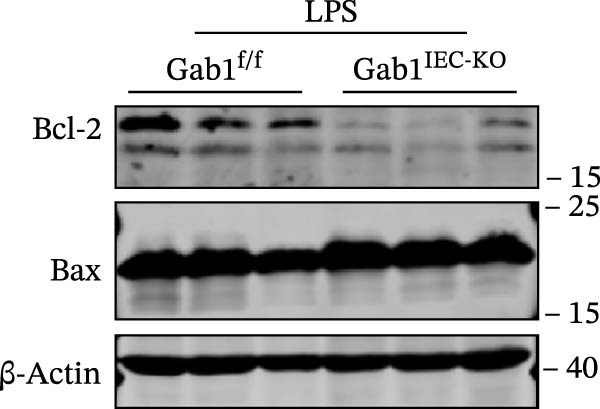
(E)

**Figure:**
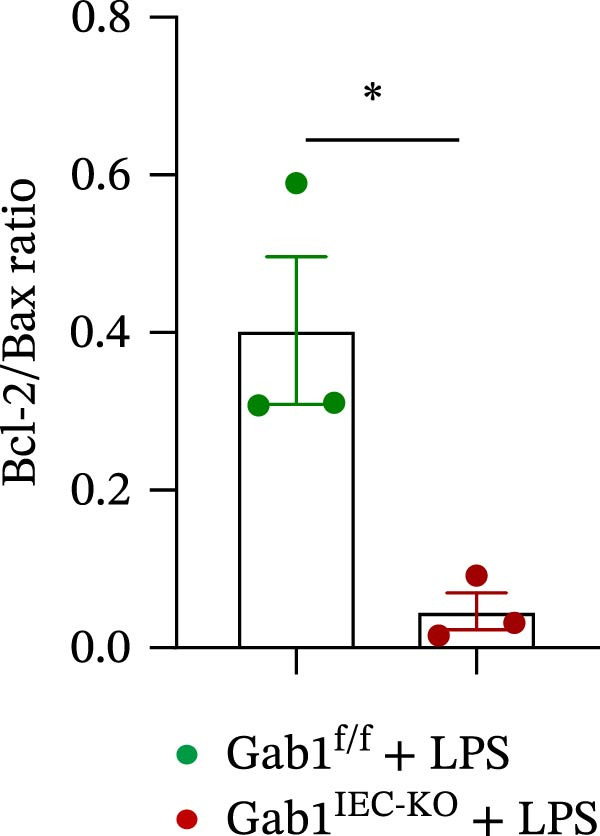
(F)

### 3.4. Gab1 Deficiency Enhances TNF‐α‐Induced IEC Apoptosis

Numerous clinical and experimental studies have identified TNF‐α as the principal mediator of sepsis pathogenesis and a key responder toward LPS challenge [41–43]. To further explore whether the exacerbated sepsis phenotype in Gab1^IEC-KO^ mice was associated with TNF‐α‐mediated IEC apoptosis, we employed the HT29 cell line treated with TNF‐α as an established in vitro model for apoptotic studies. First, our data revealed that TNF‐α stimulation significantly suppressed *Gab1* expression, whereas other pro‐inflammatory cytokines appeared to have relatively minor effects on *Gab1* level (Figure 5A). Moreover, Gab1 was downregulated by TNF‐α in a dose‐ and time‐dependent manner (Figure 5B,C). Next, stable Gab1‐knockdown HT29 cell line was established using lentiviral shRNA delivery and followed by puromycin selection. The knockdown efficiency of Gab1 was confirmed by Western blot analysis (Figure 5D). Cells were challenged with TNF‐α (100 ng/mL) and subsequently stained with Annexin V‐FITC/7‐AAD for apoptosis assessment. Flow cytometry analysis revealed a significant increase in apoptotic cells in Gab1‐knockdown (shGab1) HT29 cells upon TNF‐α treatment (Figure 5E,F). Conversely, TNF‐α‐triggered apoptosis was notably restrained by the overexpression of Gab1 (Supporting Information 2: Figure S2). Together, these results reveal that Gab1 plays a protective role in safeguarding IECs from TNF‐α‐triggered apoptotic cell death.

Figure 5Gab1 ablation exacerbates IEC apoptosis triggered by TNF‐α. (A) HT29 cells were treated with TNF‐α (20 ng/mL), IL‐1β (20 ng/mL), IL‐6 (20 ng/mL) or IFN‐γ (20 ng/mL) for 24 h, and *Gab1* mRNA level was determined by QPCR. *n* = 4 for each group. (B) *Gab1* mRNA expression in HT29 cells treated with TNF‐α (1, 5, or 20 ng/mL) for 24 h. *n* = 3 for each group. (C) Western blot for Gab1 expression in HT29 cells treated with TNF‐α (20 ng/mL) for indicated time. β‐actin was used as a loading control. (D) Western blot for Gab1 expression in HT29 cells infected with Control, shGab1‐(1) or shGab1‐(2) lentivirus. β‐actin was used as a loading control. (E,F) Control and Gab1‐knockdown (shGab1) HT29 cells were treated with TNF‐α (100 ng/mL) for 24 h, and then apoptotic cells were analyzed by 7‐AAD and Annexin‐V double staining. Representative flow cytometry plots (E) and corresponding quantification (F) are displayed. Data are shown as mean ± SEM and are representative of three independent experiments. Statistical significance was assessed by using 1‐way ANOVA with multiple comparisons test (A, B) and 2‐way ANOVA with multiple comparisons test (F);  ^∗^ *p* <  0.05,  ^∗∗^ *p* <  0.01,  ^∗∗∗^ *p* <  0.001. ns, not significant.

**Figure:**
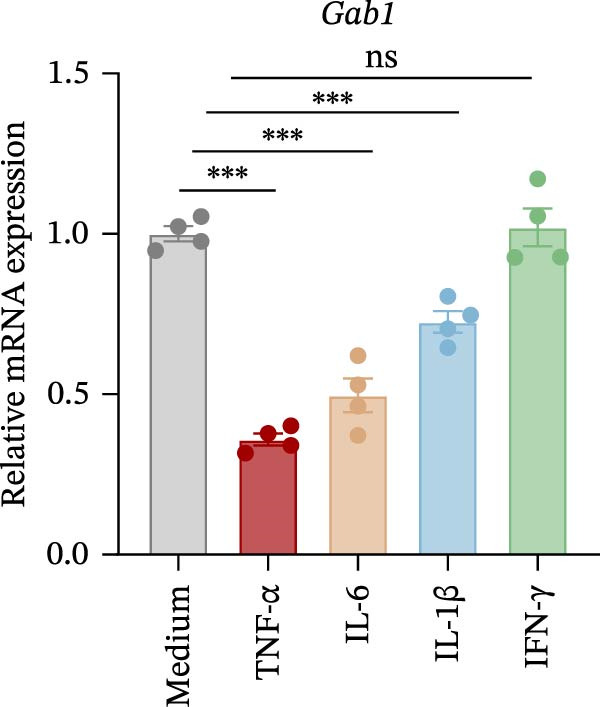
(A)

**Figure:**
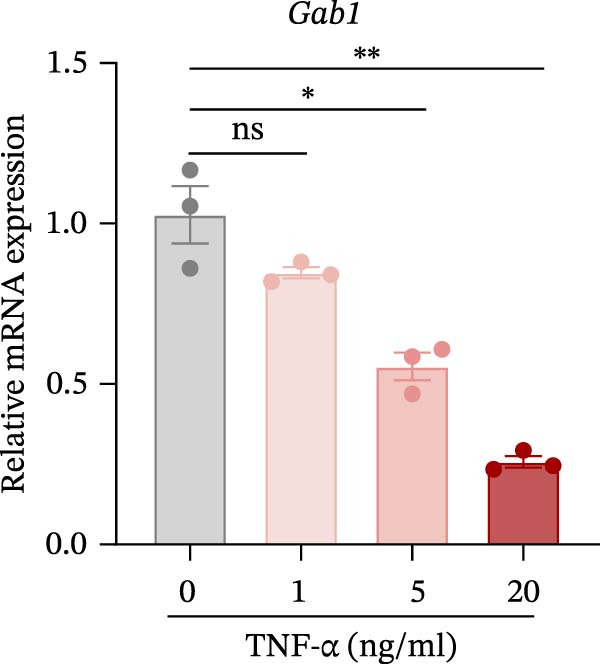
(B)

**Figure:**
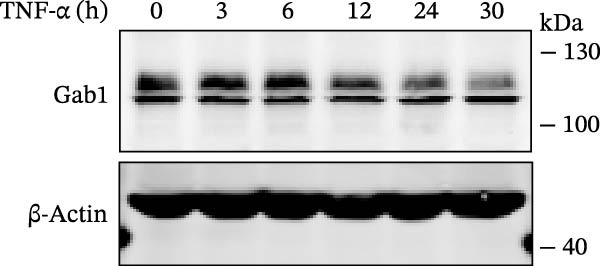
(C)

**Figure:**
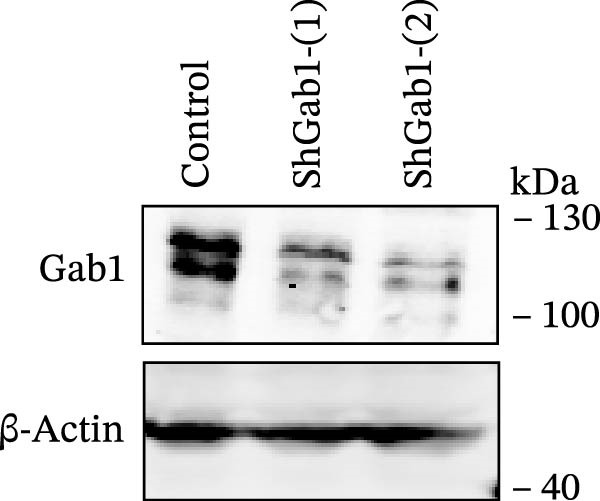
(D)

**Figure:**
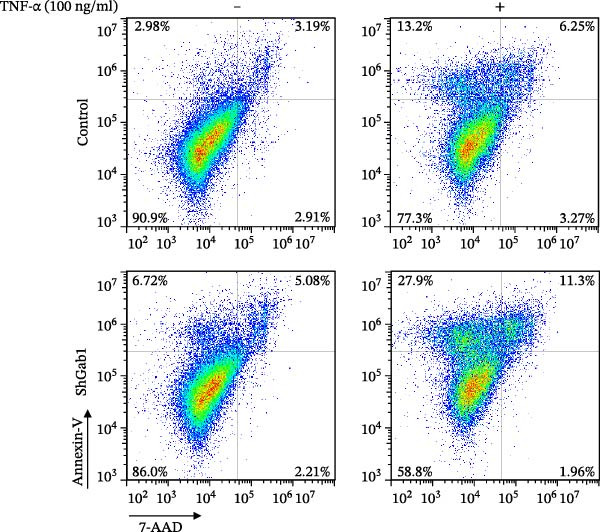
(E)

**Figure:**
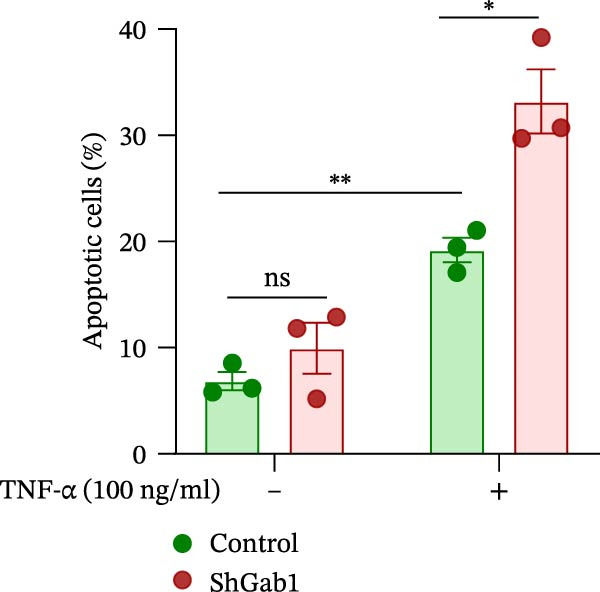
(F)

### 3.5. Gab1 Sustains Apoptotic Balance via NF‐κB Activation

Apoptosis critically contributes to sepsis pathogenesis. Both murine models and human autopsies reveal minimal histological changes in sepsis except for heightened apoptosis in gut epithelium and lymphocytes compared to non‐septic controls [13–15, 44]. Of note, preventing sepsis‐induced intestinal apoptosis significantly improves survival, by 2‐ to 10‐fold, in septic peritonitis and pneumonia. This highlights the critical importance of understanding the molecular mechanisms governing apoptosis in sepsis. The Bcl‐2 protein family, comprising anti‐apoptotic (e.g., Bcl‐2, Bcl‐XL) and pro‐apoptotic (e.g., Bax, Bak) members, crucially dictates cellular fate in response to TNF‐α stimulation. As shown in Figure 6A, Gab1 ablation shifted the balance of Bcl‐2 family proteins towards apoptosis after TNF‐α treatment, characterized by decreased levels of Bcl‐2 and Bcl‐XL, as well as increased Bax expression. Quantitatively, this finding was confirmed by a significant decrease in Bcl‐2/Bax and Bcl‐xL/Bax ratios (Figure 6B). Consistent with this, Gab1 deficiency led to a marked increase in cleaved caspase‐3 and ‐8 in response to TNF‐α treatment, indicating that the deletion of Gab1 enhances caspase activation, thus promoting apoptosis (Figure 6C).

Figure 6Gab1 maintains anti‐apoptotic protein expression by promoting p65 nuclear translocation via binding with IKKβ. (A,B) Control (Scr) and Gab1‐knockdown (shGab1) HT29 cells were treated with TNF‐α (20 ng/mL) for 24 h, and the expression of Bcl‐2, Bax, and Bcl‐XL was determined by Western blotting (A). Quantitative data was shown as mean ± SEM for 3 independent experiments (B). (C) Control and Gab1‐knockdown HT29 cells were treated with TNF‐α (20 ng/mL) for 6 or 24 h. The expression of pro‐ and cleaved‐Caspase 3, pro‐ and cleaved‐Caspase 8 was determined by Western blotting. (D) Control and Gab1‐knockdown HT29 cells were treated with TNF‐α (20 ng/mL) for indicated time, and phosphorylation of p65, ERK, and p38 was determined by Western blotting. (E) Immunofluorescence staining of control (Scr) and Gab1‐knockdown (shGab1) HT29 cells after exposure to TNF‐α (20 ng/mL) for 30 min (red, p65; blue, DAPI; scale bars, 20 μm). Quantitative analysis of the ratio of nuclear/cytoplasmic fluorescence intensity was shown as mean ± SEM. *n* = 3, 3, 10, 10 for each group. (F) Control (Scr) and Gab1‐knockdown (shGab1) HT29 cells were stimulated with TNF‐α (20 ng/mL) for 30 min. And the indicated proteins in cytoplasmic extracts and nuclear extracts were measured by Western blotting. Lamin B1 (nuclear fraction), GAPDH and β‐tubulin (cytoplasmic fraction) were used as loading controls. (G) Luciferase reporter assay for NF‐κB transcriptional activity. HEK293T cells were co‐transfected with pNF‐κB‐Luc and pRL‐TK plasmids, along with Scr or shGab1 plasmids, followed by stimulation with TNF‐α (20 ng/mL) for 8 h before being harvested for luciferase assay. (H) Control and Gab1‐knockdown HT29 cells were stimulated with TNF‐α (20 ng/mL) for 2 and 5 min. The levels of p‐IKKα/β, IKKα, and IKKβ was determined by Western blotting. (I) HEK293T cells were co‐transfected with Gab1‐Flag and IKKβ‐HA for 24 h. Cell lysates were then immunoprecipitated using anti‐flag antibody and analyzed by immunoblotting with anti‐HA antibody. Data are shown as mean ± SEM and are representative of three independent experiments. Statistical significance was assessed by using 2‐tailed Student’s *t* test (B) and 2‐way ANOVA with multiple comparisons test (E, G);  ^∗∗∗^ *p* <  0.001,  ^∗∗∗∗^ *p* <  0.0001.

**Figure:**
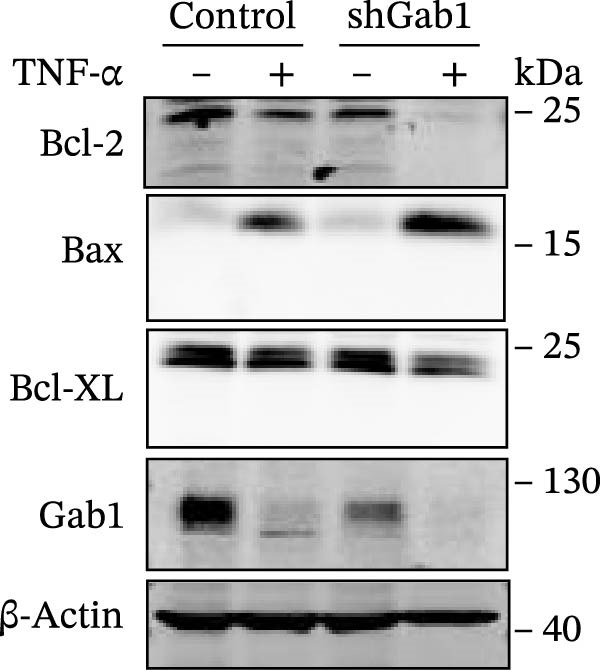
(A)

**Figure:**
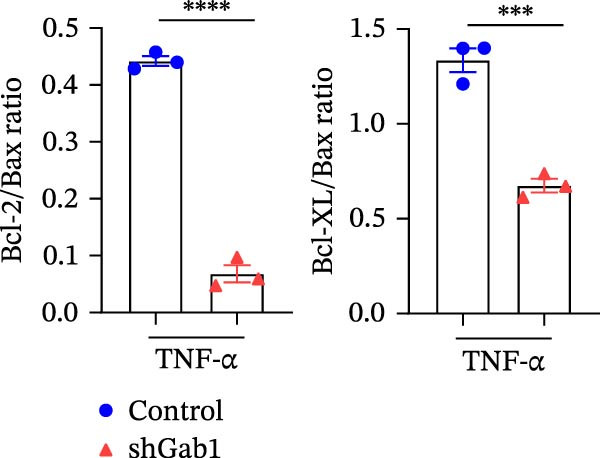
(B)

**Figure:**
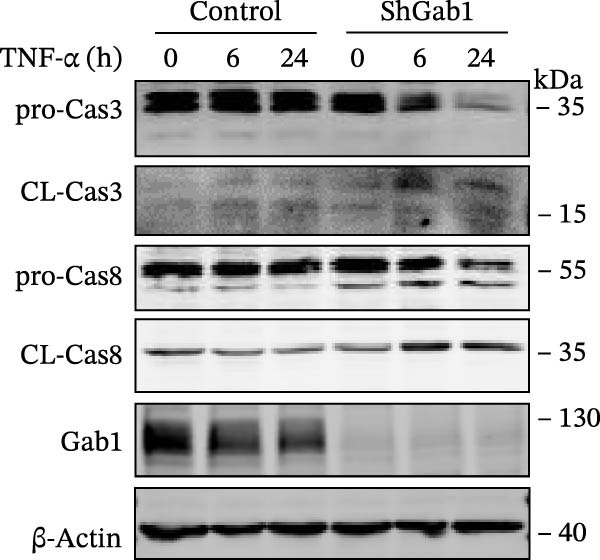
(C)

**Figure:**
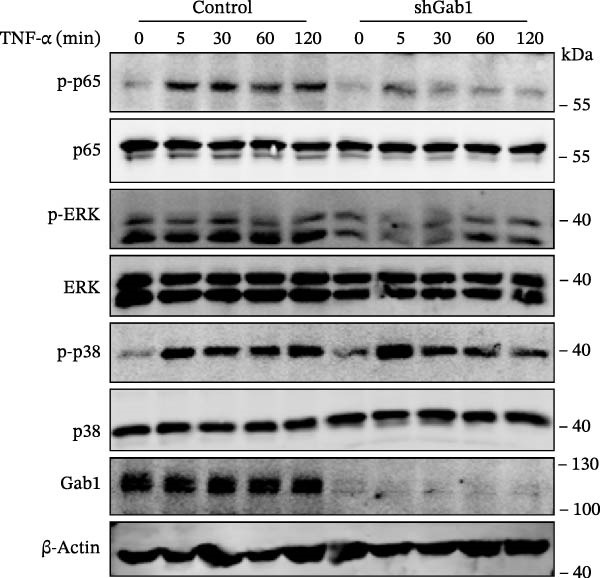
(D)

**Figure:**
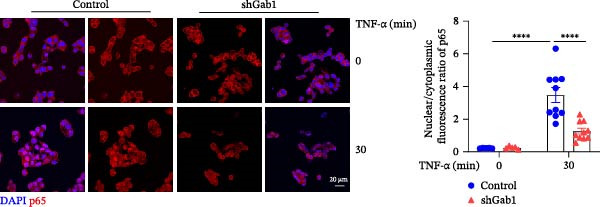
(E)

**Figure:**
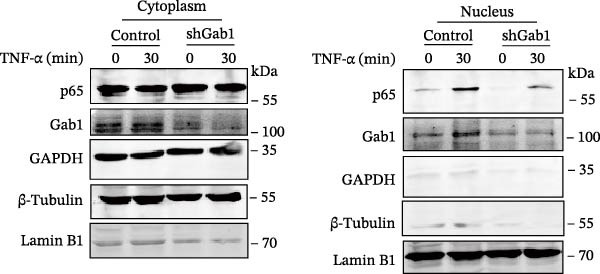
(F)

**Figure:**
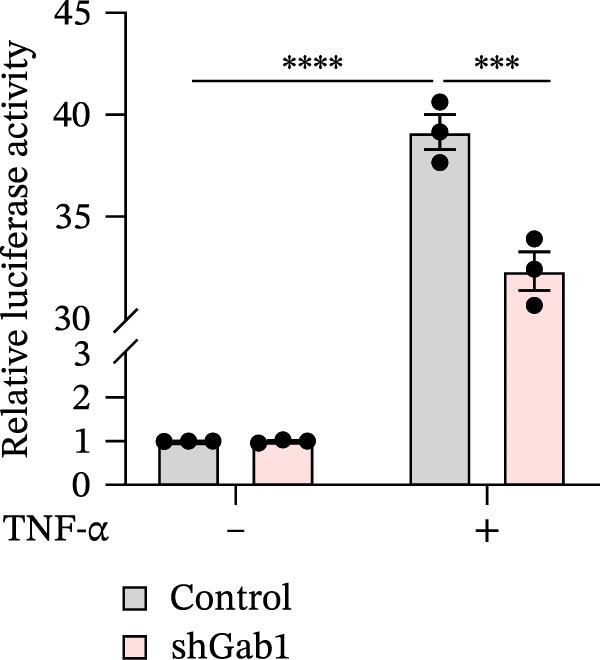
(G)

**Figure:**
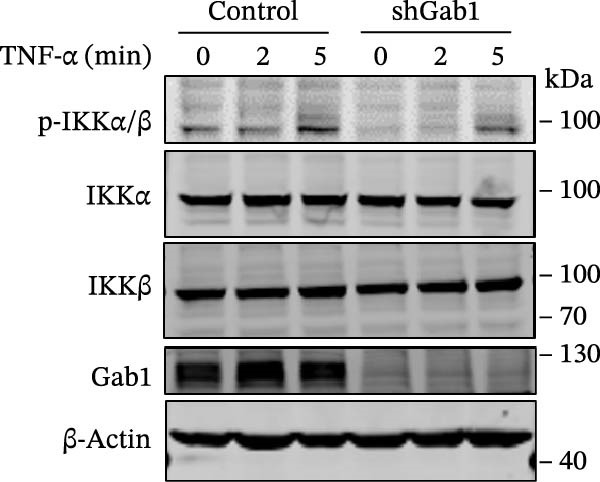
(H)

**Figure:**
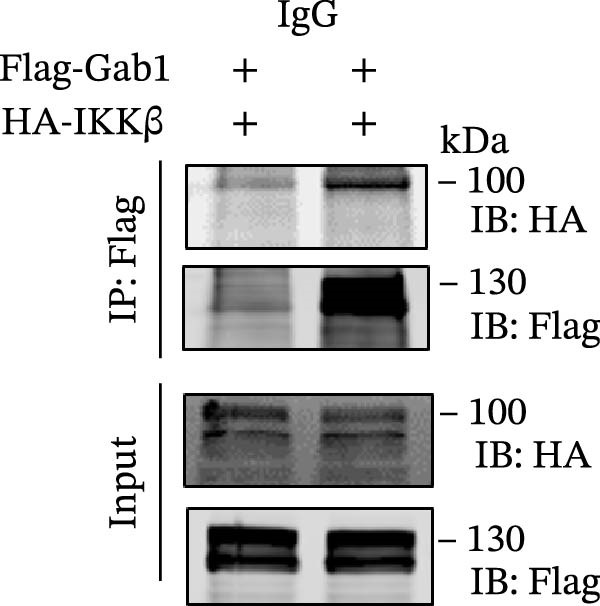
(I)

NF‐κB activation protects cells from TNF‐α‐mediated apoptosis by inducing anti‐apoptotic proteins [45, 46]. This protective mechanism involves TNF binding to TNFR1, thereby activating the IKK complex, which leads to IκB degradation and facilitates the nuclear translocation of p65 [47]. Nuclear‐localized p65 transcriptionally regulates numerous NF‐κB target genes, including key anti‐apoptotic Bcl‐2 family members. However, the dysregulation of Bcl‐2 family balance, which promotes Bax activation and Bcl‐2 suppression, can lead to aggravated apoptosis in septic epithelia. Western blot analysis showed that the phosphorylation level of p65 was remarkably attenuated in Gab1‐knockdown HT29 cells following TNF‐α treatment, while the activation of ERK1/2 and p38 remained unaffected (Figure 6D). Next, we examined the subcellular location of p65 under TNF‐α stimulation. Following 30 min of TNF‐α exposure, immunostaining revealed increased nuclear accumulation of p65 in HT29 cells, whereas Gab1 deficiency suppressed the nuclear translocation of p65 (Figure 6E). To confirm this observation, cells were fractionated into cytoplasmic and nuclear components and assessed for relative p65 levels. As shown in Figure 6F, TNF‐α exposure shifted p65 into the nucleus, and notably, the deletion of Gab1 substantially blocked this nuclear accumulation. Consistent with these findings, the NF‐κB luciferase reporter assay revealed that TNF‐α‐induced transcriptional activity was markedly reduced upon Gab1 knockdown (Figure 6G), further confirming the suppression of NF‐κB signaling at the transcriptional level. To explore the underlying mechanism by which Gab1 positively regulates NF‐κB activation, we evaluated the phosphorylation of the IKK complex. Western blot analysis revealed that Gab1 knockdown led to a reduction in p‐IKKα/β levels following rapid TNF‐α stimulation (Figure 6H). We further tested for a potential physical association via co‐immunoprecipitation assays; notably, Gab1 was found to interact with IKKβ in transfected HEK293T cells (Figure 6I). These results demonstrate that Gab1 interacts with IKKβ to facilitate IKK activation and NF‐κB transcription, thereby protecting IECs from TNF‐α‐induced apoptosis.

In summary, our findings underscore a protective role for Gab1 in alleviating sepsis‐induced intestinal injury and inflammation by targeting the IKK/NF‐κB signaling axis to suppress epithelial cell apoptosis, ultimately improving survival outcomes.

## 4. Discussion

Sepsis is now characterized by the coexistence of simultaneous hyperinflammation and immune suppression, a complex process orchestrated by the interplay of pathogenic, host, and environmental factors [10, 48]. While the current treatment approach focuses on the management of infection and restoration of perfusion, there remains a notable absence of targeted therapies to address specific forms of host dysregulation, such as impairments in gut and vascular permeability [2].

Increasing evidence demonstrated that intestinal barrier dysfunction contributes to the pathogenesis of sepsis, which can lead to subsequent profound tissue damages and multiorgan failure by driving local and systemic inflammation through diverse feedback loops [4, 49]. Clinical evidence highlighted intestinal hyperpermeability occur in critically ill patients, and this positively correlates with multiple organ failure and poor prognosis [50, 51]. Furthermore, animal studies indicated that both a leaky gut barrier and gut microbiota dysbiosis are intrinsic to sepsis [52]. Therefore, it is of great importance to unravel the underlying mechanisms that maintain intestinal barrier homeostasis and mitigate septic injury throughout the sepsis progression. In the current study, we revealed a marked reduction of Gab1 within the intestine in both sepsis patients and several established sepsis models. Further RNA‐Seq analysis identified that Gab1 is downregulated in IECs, suggesting the probable role of epithelial Gab1 in septic injury and disease progression.

Serving as an adaptor protein, Gab1 plays a central role in integrating and amplifying the downstream signaling cascades initiated by growth factors or cytokines [27–29, 53]. Global Gab1 knockout mice are embryonic lethal because of multiple defects in placenta, heart, skin, and muscle [30]. Although Gab1 is ubiquitously expressed, its function exhibits distinctly tissue‐ and cell‐type specificity. Emerging evidence highlights Gab1’s role in regulating tissue damage and organ fibrosis, particularly in hepatocytes and cardiomyocytes, primarily through its modulation of apoptosis within these cells [35, 36, 54]. Despite these insights, the specific role of intestinal Gab1 during sepsis‐induced intestinal damage and its contribution to septic lethality remain to be elucidated. LPS, a major component of Gram‐negative bacterial outer membrane, is the most potent microbial trigger in sepsis pathogenesis [55, 56]. As an established model for murine sepsis, LPS activates Toll‐like receptor 4 (TLR4), which in turn leads to receptor dimerization and a subsequent signaling cascade. This triggers the production of pro‐inflammatory cytokines like TNF‐α, IL‐1β, and interferons, thereby driving the inflammatory and immune responses central to bacterial sepsis [57]. Considering the reduction of Gab1 in IECs we have demonstrated earlier, we utilized Gab1^IEC-KO^ mice to establish an LPS‐induced sepsis model and assess the role of epithelial Gab1 in sepsis progress. Notably, epithelial Gab1‐deficient mice developed more severe intestinal damage and elevated sepsis mortality following LPS challenge, accompanied by a significant increase in epithelial cell apoptosis in the Gab1^IEC-KO^ group as revealed by immunofluorescence staining.

Epithelial homeostasis relies on a delicate balance between cell proliferation and death, while uncontrolled increase of IEC death promotes gut injury, intestinal barrier dysfunction and ultimately results in a series of pathological processes [9, 58, 59]. The regulation of IEC death is highly context‐dependent, as the forms of cell death that IECs undergo vary among different diseases. Autopsies of patients with sepsis showed widespread apoptosis in epithelial cells of colon and ileum [13], which was further supported by animal studies [14, 15, 60]. In line with these observations, genetic approaches demonstrated that ablation of genes such as Tnfr1, IKKβ, and Cnlp, resulted in worsened sepsis and higher mortality, driven by aberrant IEC apoptosis [15, 16, 22]. In contrast, gut‐specific overexpressing Bcl‐2 or administering interventions that target the inhibition of IEC apoptosis and restoration epithelial barrier integrity significantly improved the outcomes of septic peritonitis in murine models [23, 25, 61, 62]. Therefore, although IEC apoptosis is considered immunologically silent during physiological cell turnover, aberrant apoptosis accelerates the pathological progression during sepsis and MODS, leading to impaired intestinal barrier function, exacerbated dysbiosis and systemic inflammation, and potentially secondary necrosis. TNF‐α is a central inflammatory cytokine that is responsible for IEC apoptosis and tissue damage in sepsis [18, 19]. Sensing of TNF by TNFR1 initiates the formation of a primary membrane‐bound receptor signaling complex (Complex I) and the subsequent activation of MAPK and canonical NF‐κB pathway. Upon this activation, nuclear NF‐κB acts as a checkpoint for cell fate by transcriptionally upregulating prosurvival genes, especially Bcl‐2 family proteins, Inhibitor of Apoptosis Proteins (IAPs) [63]. Consistent with the in vivo observations from the sepsis model described above, Gab1 deficiency markedly enhances TNF‐α‐induced apoptosis in HT29 cells. Interestingly, Gab1 knockdown has no effect on TNF‐α‐induced ERK1/2 and p38 phosphorylation, whereas p65 activation is significantly diminished compared with control group. Mechanistically, our study reveals, for the first time, a novel molecular scaffold wherein Gab1 interacts with the IKK complex, providing a necessary bridge for efficient NF‐κB activation in response to inflammatory stimuli. By facilitating the nuclear translocation of p65 and subsequent transcription of Bcl‐2 family proteins, Gab1 serves as a pivotal safeguard against IEC apoptosis in septic epithelia.

While these findings provide mechanistic insights, certain limitations regarding the experimental models should be noted. Consistent with previous studies [64, 65], the LPS‐induced sepsis model was used to achieve a high‐resolution dissection of the Gab1‐NF‐κB signaling axis. However, it should be noted that mimicking the dynamic progression and late‐stage immune dysregulation of clinical sepsis remains an inherent challenge for most animal models, including the single‐hit LPS challenge used here [2]. In addition, while the systemic LPS challenge effectively simulates the cytokine storm, it may not fully capture the continuous host–microbiota interaction and polymicrobial dysbiosis during sepsis, which is better represented in the CLP bacterial sepsis model [5, 66]. Nevertheless, the LPS model remains highly advantageous for investigating how the intestinal epithelium preserves and repairs the barrier during the hyperacute cytokine storm, the most critical and life‐threatening phase of sepsis. Our findings thus provide a critical mechanistic foundation for understanding mucosal resilience during sepsis progression.

Given the broad heterogeneity of sepsis, there is an emerging need for both predicting individualized treatment effects and developing targeted therapies to address distinct forms of host dysregulation to improve bedside management. Here, we demonstrate that Gab1 significantly protects against sepsis‐induced intestinal injury and enhances sepsis survival by orchestrating the apoptotic/anti‐apoptotic balance in IECs and maintaining intestinal barrier integrity. These findings not only deepen our understanding of sepsis pathogenesis but also identify Gab1‐mediated mucosal protection as a potential cornerstone for personalized therapeutic strategies. Ultimately, restoring the intestinal barrier through such targeted approaches holds promise for improving clinical outcomes for patients facing the formidable challenges during sepsis progression.

## 5. Conclusion

Our study definitively delineates a crucial role for Gab1 in protecting against sepsis‐induced intestinal injury. By precisely fine‐tuning NF‐κB‐mediated apoptotic signaling, Gab1 safeguards IECs, thereby preserving intestinal barrier integrity and mitigating inflammation during sepsis. These findings provide deeper insights into sepsis pathogenesis and highlight Gab1 as a promising therapeutic target for novel interventions aimed at restoring intestinal barrier function and improving outcomes in septic patients.

**Nomenclature**
IECs: Intestinal epithelial cells
MODS: Multi‐organ dysfunction syndrome
LPS: Lipopolysaccharide
CLP: cecal ligation and puncture
TNF: Tumor necrosis factor
FADD: Fas‐associated death domain protein
TRAF2: TNF Receptor‐Associated Factor 2
Gab1: Grb2‐associated binder 1
Gab2: Grb2‐associated binder 2
ALI: Acute lung injury
GBS: Group B streptococcus
UC: Ulcerative colitis
CD: Crohn’s disease
TLR4: Toll‐like receptor 4
IAPs: Inhibitor of apoptosis proteins.

## Author Contributions

Xue Zhang, Jiaqi Xu, and Wei Jin conceived the study and designed the experiments. Wei Jin and Yanchuang Wu performed most of the experiments, assisted by Jiaqi Xu. Yu Pan and Lifeng He were in charge of recruiting patients/controls and collected tissue samples. Xiaoqing Cheng performed bioinformatics analysis. Wei Jin and Xue Zhang wrote the manuscript and designed figures. Xue Zhang, Jiaqi Xu, and Yun Xu provided funding, technical, and material support. Jiaqi Xu and Yun Xu edited the manuscript.

## Funding

This work was supported by the "Pioneer" and "Leading Goose" R&D Program of Zhejiang under Grant 2024C03173 to Songmei Lou; Huadong Medicine Joint Funds of the Zhejiang Provincial Natural Science Foundation of China under Grant LHDMY24H070001 to Jiaqi Xu.

## Disclosure

All the authors have read and approved the final version of the manuscript.

## Conflicts of Interest

The authors declare no conflicts of interest.

## Supporting Information

Additional supporting information can be found online in the Supporting Information section.

## Supporting information


**Supporting Information 1** Supporting Information 1 Table S1. Basic information of clinical samples. Table S2. RT‐qPCR primer sequences.

**Supporting Information 2** Supporting Information 2 Figure S1. Gab2 expression remains unchanged in sepsis‐induced intestinal injury. Figure S2. Gab1 overexpression suppresses IEC apoptosis induced by TNF‐α.

## Data Availability

The data that support the findings of this study are available from the corresponding author upon reasonable request.
