# Supplementary material for: Epithelial Gab1 Restricts Sepsis‐Induced Intestinal Injury by Orchestrating TNF/NF‐κB Axis
Source: Mediators Inflamm. 2026 Jan 31;2026:5486971. doi: 10.1155/mi/5486971 (PMC12860144; doi:10.1155/mi/5486971)
Supplement: Supplementary file 1 — Supporting Information 1 Supporting Information 1 Table S1. Basic information of clinical samples. Table S2. RT‐qPCR primer sequences. [file MI-2026-5486971-s002.docx]

**Supplementary Table 1. Basic Information of Clinical Samples**

| **Characteristics** | Control  (n=7) | Sepsis  (n=7) | | CD-induced sepsis (n=7) |
| --- | --- | --- | --- | --- |
| **Age, Year** (mean ± SD) | 71.7 ± 2.9 | 66.0 ± 12.6 | | 57.7 ± 18.1 |
| Male / Female n (%) | 3 (42.9%) / 4 (57.1%) | 5 (71.4%) / 2 (28.6%) | | 4 (57.1%) / 3 (42.9%) |
| **APACHE Ⅱ Score** (mean ± SD)  **CRP** (mean ± SD)  **PCT** (mean ± SD) | NA  1.3±0.9  NA | 26.0± 11.9  77.8±49.1  48.6±40.5 | NA  71.5±67.5  NA | |
| **ESR** (mean ± SD) | NA | NA | 30.6±22.5 | |
| **ICU length of stay, Day**  (mean ± SD) | NA | 7.9±4.6 | NA | |

APACHE Ⅱ score, Acute Physiology and Chronic Health Evaluation II Score; CRP, C-reactive protein; PCT, procalcitonin; ESR, erythrocyte sedimentation rate; NA: not-available.

**Supplementary Table 2. RT-qPCR Primer Sequences**

| **Gene** | **Forward Primer (5’ to 3’）** | **Reverse Primer (5’ to 3’）** |
| --- | --- | --- |
| **Mouse Genes** | | |
| *Il-6* | AGTTGCCTTCTTGGGACTGA | TCCACGATTTCCCAGAGAAC |
| *Il-1β* | TGTGGCTGTGGAGAAGCTGT | CAGCTCATATGGGTCCGAGA |
| *Tnf-α* | CTGGGACAGTGACCTGGCT | GCACCTCAGGGAAGAGTCTG |
| *Ccl2* | AACTCTCACTGAAGCCAGCTCT | CGTTAACTGCATCTGGCTGA |
| *β-actin* | AACAGTCCGCCTAGAAGCAC | CGTTGACATCCGTAAAGACC |
| **Human Genes** |  |  |
| *Gab1* | CTACCTGTTGCTCATCAACTGT | GGGACGTTATCATTGCAGTCTG |
| **­***β-actin* | AAGGTGAAGGTCGGAGTCAAC | GGGGTCATTGATGGCAACAATA |
|  |  |  |
