# Supplementary material for: Epithelial Gab1 Restricts Sepsis‐Induced Intestinal Injury by Orchestrating TNF/NF‐κB Axis
Source: Mediators Inflamm. 2026 Jan 31;2026:5486971. doi: 10.1155/mi/5486971 (PMC12860144; doi:10.1155/mi/5486971)
Supplement: Supplementary file 2 — Supporting Information 2 Supporting Information 2 Figure S1. Gab2 expression remains unchanged in sepsis‐induced intestinal injury. Figure S2. Gab1 overexpression suppresses IEC apoptosis induced by TNF‐α. [file MI-2026-5486971-s001.doc]

**
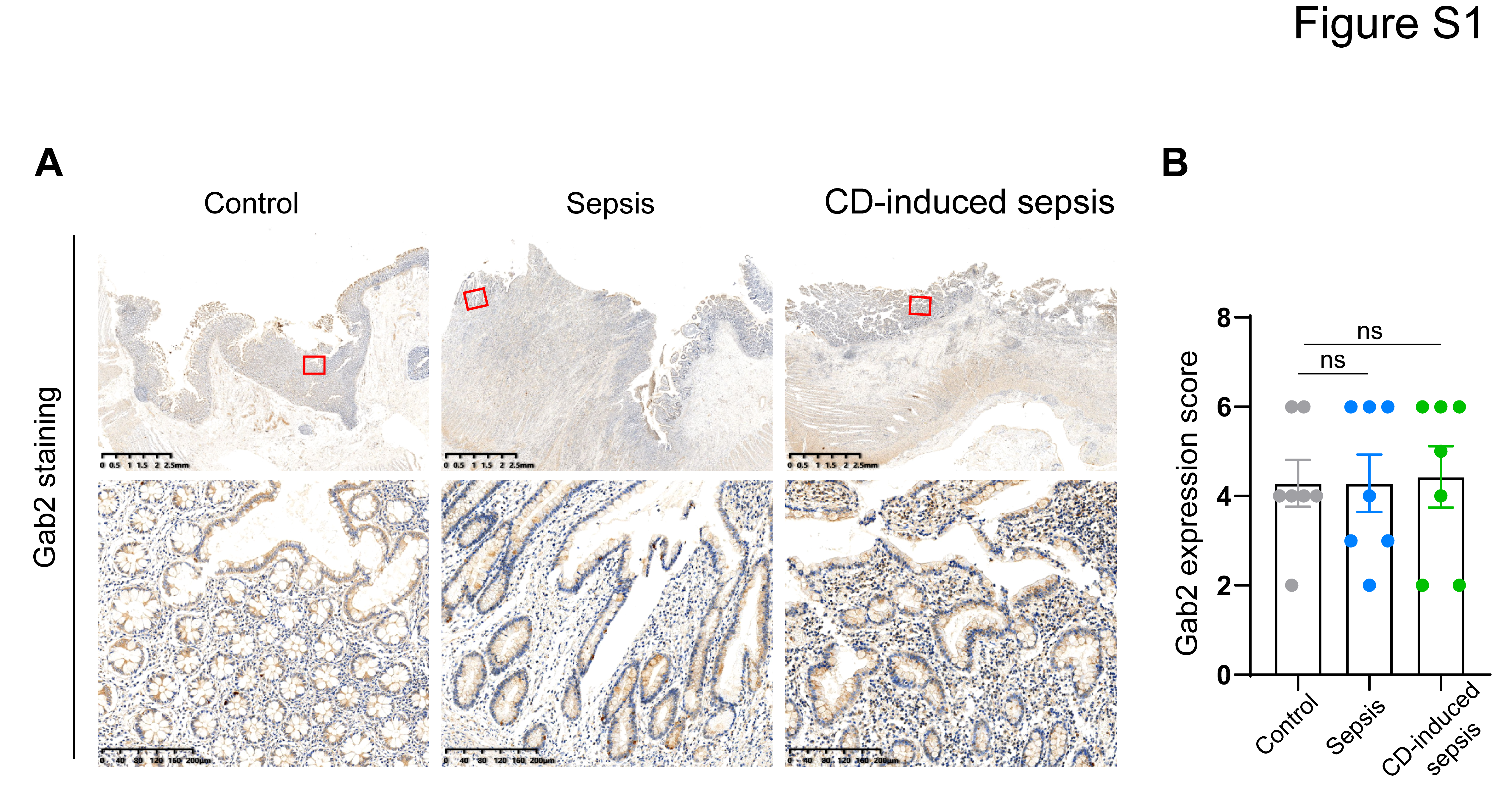
**

**Supplementary Figure 1. Gab2 expression remains unchanged in sepsis-induced intestinal injury.**

**(A-B)**Representative IHC staining (A) and quantitative analysis (B) of Gab2 expression in intestines from patients with sepsis (non-CD sepsis) or CD-induced sepsis, and normal controls. *n* = 7 for each group. Scale bars, 2.5 mm (overview) and 200 μm (magnification). Quantitative data are shown as mean ± SEM. Statistical significance was assessed by using 1-way ANOVA with multiple comparisons test; ns, not significant.

**
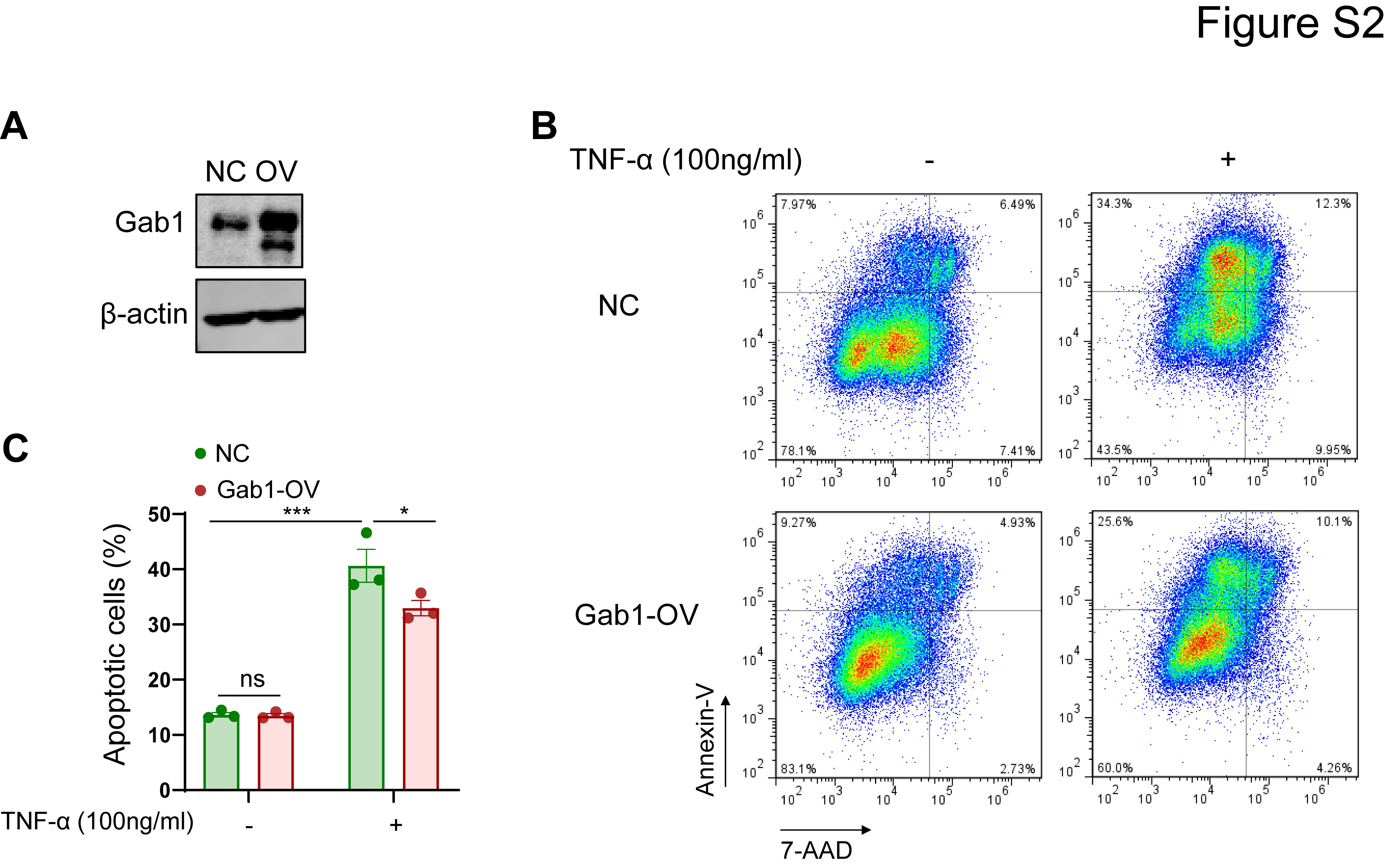
**

**Supplementary Figure 2. Gab1 overexpression suppresses IEC apoptosis induced by TNF-α.**

**(A)**Western blot for Gab1 expression in HT29 cells infected with NC (negative control) or Gab1-overexpression lentivirus. β-actin was used as a loading control.

**(B-C)**Control or Gab1-overexpressed HT29 cells were stimulated with TNF-α (100 ng/mL) for 48 hours. Apoptotic cells were then analyzed by 7-AAD and Annexin-V double staining (B). Quantitative data was shown as mean ± SEM for 3 independent experiments (C).

Statistical significance was assessed by using 2-way ANOVA with multiple comparisons test; ^*^ *p* < 0.05; ^***^ *p* < 0.001.
